# Rice seedling age detection under field conditions using M-Lresnet50 with image and environmental data

**DOI:** 10.3389/fpls.2026.1732054

**Published:** 2026-03-31

**Authors:** Jiaxin Gao, Feng Tan, Zihan Zhu, Hongbo Xiang, Xue Chen, Chunyou Guo

**Affiliations:** 1 College of Agricultural Engineering, Heilongjiang Bayi Agricultural University, Daqing, China; 2 College of Information and Electrical Engineering, Heilongjiang Bayi Agricultural University, Daqing, China; 3 Information Network Center, Panjin Vocational Technical College, Panjin, Liaoning, China; 4 Publicity Department, HeilongJiang University of Technology, Jixi, China

**Keywords:** dynamic channel pruning, multimodal recognition, Resnet50, rice seedling age, row-prior strip-attention

## Abstract

**Introduction:**

Accurate identification of rice seedling age is essential for guiding precise field management and optimizing agronomic practices. However, traditional identification methods mainly rely on manual experience or simple visual cues and often lack robustness under complex field conditions such as illumination variation, background interference, and subtle morphological differences between adjacent growth stages. Therefore, developing a reliable and automated method for fine-grained recognition of rice seedling stages is of great importance.

**Methods:**

To address this problem, this study proposes two deep learning models for automatic recognition of 13 rice seedling stages. The first model, Lresnet50, enhances visual feature representation by improving the baseline Resnet50 with a Row-Prior Strip Attention (RPS) mechanism, a Feature Pyramid Network (FPN) for multi-scale feature extraction, and Dynamic Channel Pruning (DCP) to reduce redundant channels and improve computational efficiency. Based on this model, a multimodal framework named M-Lresnet50 is further developed by integrating image features with temporal environmental data through a Long Short-Term Memory (LSTM) network, enabling cross-modal feature fusion and improving recognition of continuous seedling growth stages.

**Results:**

Experimental results demonstrate that the proposed models achieve high accuracy in recognizing 13 rice seedling stages. The Lresnet50 model achieves an average classification accuracy of 97.70%, outperforming several existing convolutional neural network architectures and showing strong performance in transitional growth stages where morphological differences are subtle. By integrating visual features with temporal environmental information, the multimodal M-Lresnet50 further improves the accuracy to 98.33%. The model contains 27.656 million parameters with a computational complexity of 13.965 GFLOPs, indicating a good balance between recognition accuracy and computational cost.

**Discussion:**

The results confirm the effectiveness of the proposed improvements and multimodal fusion strategy. The Row-Prior Strip Attention (RPS) enhances the model’s ability to focus on row-structured crop regions, while the Feature Pyramid Network (FPN) improves multi-scale feature representation. In addition, Dynamic Channel Pruning (DCP) reduces redundant channels and improves computational efficiency. The integration of temporal environmental information through the multimodal framework further enhances the robustness and consistency of seedling stage recognition. Overall, the proposed approach provides a practical solution for intelligent monitoring of rice seedling growth in greenhouse environments.

## 1 Introduction

Rice, a staple crop of global importance (Fukagawa and Ziska, 2019), requires precise monitoring of its growth cycle to improve yield and optimize cultivation strategies. The age of seedlings is a key indicator of the early growth and developmental status of rice plants, and timely knowledge of seedling stage is critical for informing management decisions such as fertilization, irrigation, and pest and disease control. Early detection of deviations in seedling age or growth allows farmers to implement corrective measures promptly, reducing potential yield losses and improving resource use efficiency. Consequently, accurate and timely identification of seedling stages is not only practically important but also essential for enabling intelligent and precise agricultural management.

Conventional methods for rice seedling age identification mainly rely on manual experience or shallow visual feature analysis from two-dimensional images, such as leaf counting (Zhou et al., 2025), color thresholding (Liao et al., 2018), and morphological recognition; however, these methods are highly sensitive to field environmental variations, including illumination changes and complex backgrounds, which limits their robustness under natural conditions. In recent years, the rapid development of deep learning has enabled the application of convolutional neural networks (CNNs) and lightweight architectures to crop growth monitoring and disease diagnosis, leading to substantial performance improvements (Naresh kumar and Sakthivel, 2025; Wang et al., 2025; Zeng and He, 2024). In the context of rice-related studies, Rahman et al. (2020) developed a lightweight two-stage CNN for rice disease and pest detection, achieving over 93% accuracy with a reduced model size suitable for mobile deployment. Qin et al. (2023) proposed a Resnet50-based framework for continuous recognition of six rice phenological stages, reaching an accuracy of 87.33%. Beyond rice, Li et al. (2020) introduced a lightweight SE-Inception-based model that achieved 98.29% and 99.27% accuracy for tomato and eggplant disease recognition, respectively. Lv et al. (2022) improved YOLOv5-B with BiFPN-S and ACON-C for high-precision apple growth morphology recognition, while Duong et al. (2020) demonstrated the effectiveness of EfficientNet and MixNet on a large-scale fruit dataset of over 60,000 images. In conclusion, deep learning has become a driving force in advancing agricultural computer vision tasks, leading to breakthrough achievements in areas such as crop disease identification and fruit classification.

Furthermore, a range of specialized solutions have been proposed to address unique challenges in agricultural scenarios, such as fruit occlusion, complex backgrounds (Liu et al., 2024), and small-sample recognition (Cao et al., 2023; Lin et al., 2022). In their 2019 study, Liu et al. (2019) combined a color model with homomorphic filtering for the purpose of image enhancement. They also introduced a binocular vision-based localization method, thereby improving the robustness of kiwifruit recognition under occlusion conditions. In their 2024 study, Nakatani et al. (2024) proposed a novel integration of YOLOv5, GrabCut segmentation, Harris corner detection, and meta-learning strategies with the aim of developing an automated system for the estimation of floral arrangement structures. The researchers further enhanced the practicality of the system through the incorporation of a human–computer interaction interface. Despite the advances achieved in the field, two significant challenges persist in the identification of rice seedling age. Firstly, the field environment is characterized by its complexity and variability. Factors such as lighting, background clutter, and noise can readily lead to identification errors. Secondly, the morphological variations of rice seedlings across different growth stages are frequently imperceptible, which renders it challenging to attain high-precision classification through the utilization of static image data alone. Consequently, the development of models that can effectively integrate multi-source information and incorporate spatiotemporal perception is of paramount importance for enhancing the accuracy and robustness of rice seedling age identification.

In recent years, the rapid development of environmental sensors and Internet of Things (IoT) technologies has enabled the efficient collection of high-frequency field environmental data, such as temperature, humidity, and accumulated temperature. Previous studies have demonstrated that environmental variables are closely related to crop growth status, and that integrating image data with environmental information can significantly improve the accuracy of crop growth monitoring and prediction (Lin et al., 2023). Consequently, multimodal deep learning methods have become an important direction for advancing agricultural intelligence. In weed and crop recognition, Sun et al. (2021) integrated visible and near-infrared images to improve an R-FCN model using deformable convolutions and online hard example mining, achieving robust beet and weed identification under complex environments. Xu et al. (2025) combined RGB and depth data within a dual-path Swin Transformer, where cross-modal alignment attention effectively addressed morphological similarity and occlusion in wheat fields. In crop disease diagnosis, Deng et al. (2024) proposed RustQNet by integrating RGB, multispectral, and vegetation index data for early quantitative assessment of wheat stripe rust, while Liu et al. (2024) developed PSOC-DRCNet to improve generalization in rice disease recognition. Yu et al. (2025) introduced a dual-branch MMCG-MHA model combining GRU and CNN to extract spatiotemporal features for early detection of rice sheath blight, and Wang et al. (2024) proposed WCG-VMamba for maize disease identification using multi-visual encoders and wavelet transforms. In yield prediction and growth monitoring, Maimaitijiang et al. (2020) utilized UAV-based RGB, multispectral, and thermal imagery to enhance soybean yield prediction, while Mena et al. (2024) and Aviles Toledo et al. (2024) further integrated meteorological, soil, topographic, hyperspectral, and LiDAR data to achieve fine-scale and interpretable yield estimation. Li et al. (2023) proposed the Vision-Sensor Transformer to efficiently fuse image and sensor data across multiple crop growth prediction tasks. In water stress detection and irrigation management, multimodal approaches combining image and environmental data have achieved high-precision plant water status and soil moisture estimation (Wakamori et al., 2020; Zhang et al., 2024; Zuo et al., 2023). In phenotyping and crop classification, Zhou et al. (2024) integrated multimodal soybean phenotypic data with self-supervised contrastive learning for growth stage determination and lodging detection. Ma et al. (2025) proposed TomPhenoNet for multi-task tomato phenotyping using RGB-D images, while Bi et al. (2025) developed a lightweight dual-branch network for maize variety identification. In the field of seed detection, Li et al. (2025) developed the MSCNSVN model by integrating multi-source data, thereby achieving a substantial enhancement in the non-destructive detection accuracy of maize seed viability. In their study, Shamsuddin et al. (2024) utilized a combination of RGB images, phenotypic data, and historical meteorological data to predict maize yield potential. They employed SHAP analysis to identify the key contributing factors. Overall, multimodal deep learning has achieved notable progress in crop disease diagnosis, phenotypic analysis, and yield prediction, and is evolving towards lightweight architectures and improved generalization. However, targeted studies on rice seedling age identification, which involves continuous and subtle morphological variations, remain limited. In light of the highly dynamic and complex field environment, where variations in lighting and background noise frequently compromise model performance, there is an urgent need to construct a multimodal recognition framework that integrates both image and environmental data while incorporating spatiotemporal perception. This study aims to address this need by developing a high-performance multimodal model for precise rice seedling age identification, enhancing model robustness and practical applicability in challenging farmland scenarios.

The proposed methodology is an innovative approach to the identification of rice seedling age, which integrates multimodal data fusion to construct two deep learning models. These models leverage the complementary advantages of image visual information and environmental time-series data. Firstly, the Lresnet50 model enhances the standard Resnet50 by integrating a Row-Prior Strip Attention mechanism to leverage row-planting priors, a Feature Pyramid Network to capture multi-scale features, and a Dynamic Channel Pruning module for adaptive feature recalibration. Building on this, the M-Lresnet50 model further incorporates a Long Short-Term Memory (LSTM) network to model temporal environmental data, with a cross-modal fusion layer that jointly leverages visual and environmental features for more robust recognition. This approach has been shown to enhance the perception of subtle variations during the continuous changes of seedling age, thereby facilitating more precise and robust identification of rice seedling growth stages.

The primary contributions of this paper are as follows:

1. We proposed a lightweight Lresnet50 model, achieving an accuracy of 97.70% in the recognition of 13 fine-grained rice seedling age stages, significantly outperforming mainstream CNN methods.
2. We introduced a multimodal fusion model, M-Lresnet50, which integrates image and environmental features, further improving recognition accuracy to 98.33%.
3. Correlation analysis revealed a strong association between seedling age and accumulated temperature as well as effective accumulated temperature, confirming the critical role of heat accumulation in rice growth and development.
4. Generalization experiments demonstrated the model’s robustness across different times, locations, and rice varieties, highlighting its potential for applications in intelligent agricultural management.

Extensive experimentation has been conducted to assess the efficacy of the proposed method in comparison to existing mainstream approaches. The experimental results demonstrate that the proposed method exhibits superior performance in terms of identification accuracy, robustness, and generalizability.

## 2 Materials and methods

### 2.1 Data sources

#### 2.1.1 Image data collection

**Figure 1:**
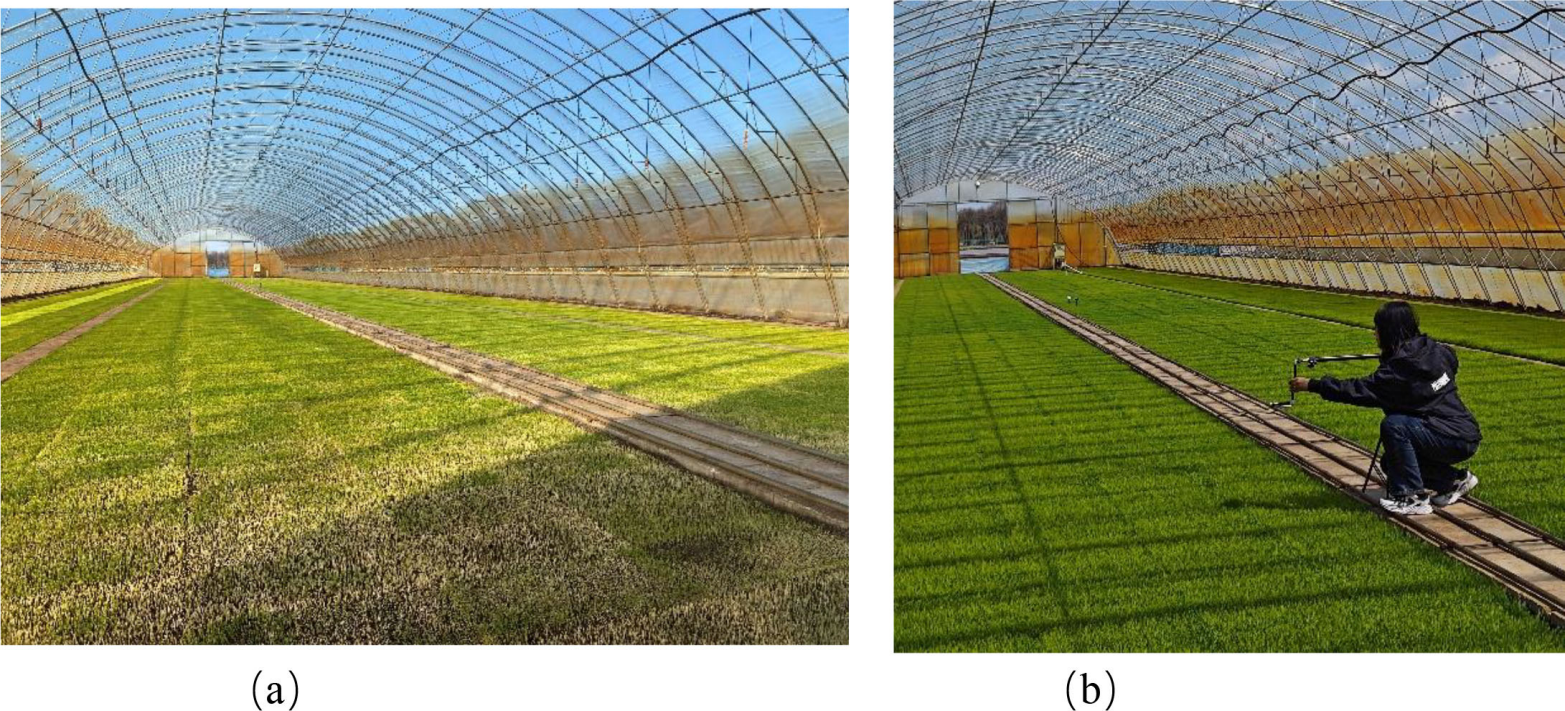
Experimental area. **(a)** Experimental site, **(b)** Data acquisition.

The experiment was conducted at the Great Northern Wilderness Xu Yirong High-Tech Rice Demonstration Park in Hulin City, Jixi City, Heilongjiang Province, China. The central geographic coordinates of the site are 132°42′51″E and 45°37′27″N, and the elevation is approximately 79.7 m. The region is located in a temperate continental monsoon climate zone. The experimental base is shown in **Figure 1**. The rice experimental area consisted of eight greenhouse sheds, each measuring 60 m × 12 m. Four rice varieties were selected for the study, namely Longjing 31, Xingjing 10, Kenchuan 102, and Longken 2021. These varieties are suitable for transplanting or direct seeding cultivation in the third accumulated temperature zone of Heilongjiang Province. Data collection was carried out from 12 April to 18 May 2024. To ensure spatial consistency, 458 fixed sampling points were established within the greenhouses, and images were captured daily at the same locations. Image acquisition was performed using a Xiaomi 13 smartphone equipped with a CMOS sensor with a maximum aperture of f/1.8. The camera was mounted on a tripod at a height of 0.6 m above the ground and oriented downward. All images were captured at a resolution of 4096 × 3072 pixels under natural lighting conditions between 9:00 and 11:00 in the morning to minimize illumination variability.

Rice seedling leaf age was determined according to the agronomic five-point method. When the emerging leaf tip of the youngest leaf was visible but did not exceed one-third of the length of the fully expanded leaf below, the value was recorded as 0.1. When the length exceeded one-third but was less than one-half, it was recorded as 0.3. When it exceeded one-half but was less than two-thirds, it was recorded as 0.5. When it exceeded two-thirds but was still shorter than the fully expanded leaf below, it was recorded as 0.7. When the emerging leaf exceeded the length of the fully expanded leaf below, it was recorded as 0.9. Based on this quantitative scoring rule, rice seedlings were categorized into 13 discrete leaf-age stages, including 1.1-leaf, 1.3-leaf, 1.5-leaf, 1.7-leaf, 1.9-leaf, 2.1-leaf, 2.3-leaf, 2.5-leaf, 2.7-leaf, 2.9-leaf, 3.1-leaf, 3.3-leaf, and 3.5-leaf stages.

#### 2.1.2 Multimodal dataset source

**Figure 2:**
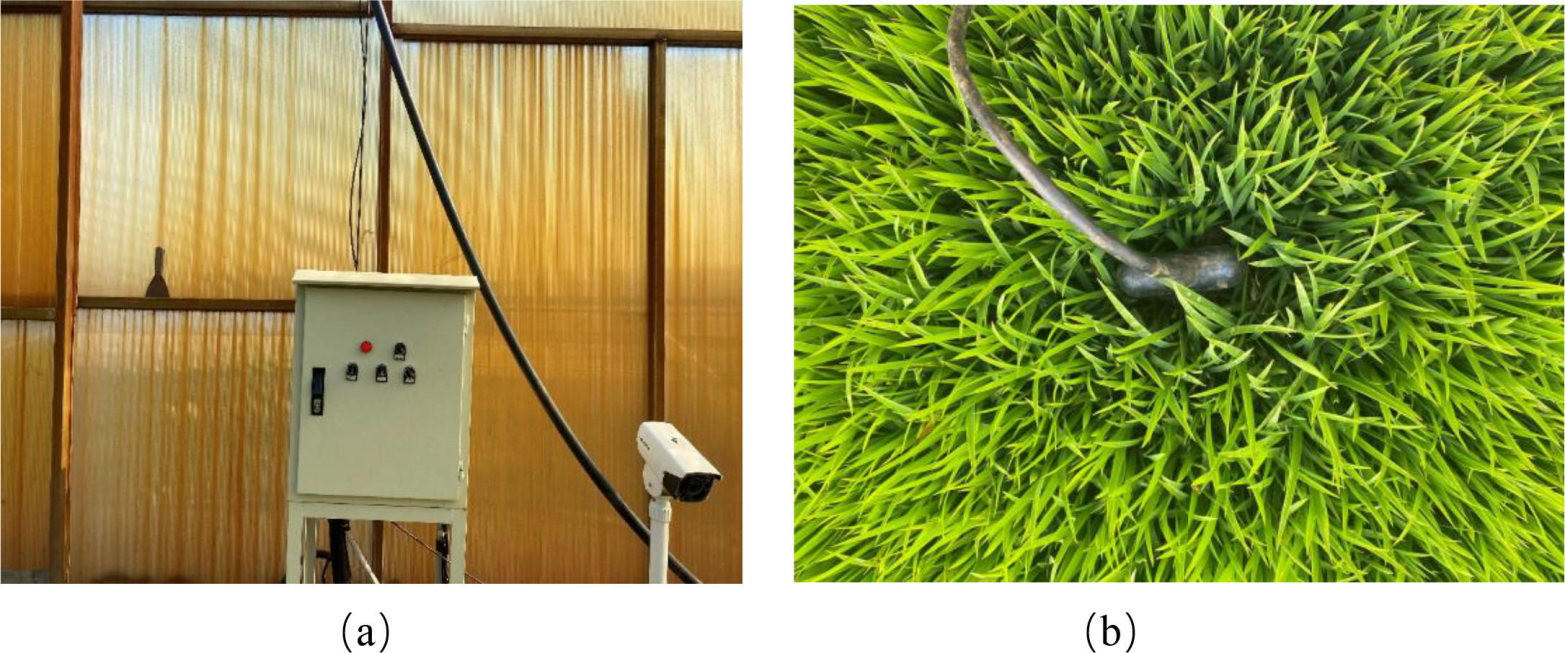
Environmental data collection device. **(a)** Environmental data collection facilities, **(b)** Soil temperature and humidity sensor.

In this study, environmental factor data were collected using multifunctional IoT sensors installed in the field, as illustrated in **Figure 2**. These sensors were designed to continuously record key indicators of the rice growth environment, including temperature, air humidity, and soil moisture. The devices were equipped with an embedded data logging system capable of automatic sampling at fixed intervals, with real-time monitoring results displayed on a built-in screen. All collected data were stored with timestamps as indices to ensure precise alignment with the corresponding image acquisition times. The environmental monitoring terminals transmitted the collected data to the on-site workstation via a serial communication interface and a wireless local area network (WLAN). The data were then further uploaded to a remote database using 5G communication technology, enabling remote storage and management of field environmental information. Furthermore, the devices were capable of facilitating the periodic export of data files, which contained timestamps and corresponding environmental parameter records. This capability enabled subsequent batch analysis and modelling. In order to ensure synchronization with the image data, the image acquisition time was utilized as a calibration anchor point. During the process of data preprocessing, a timestamp-based matching or interpolation technique was employed to align the environmental feature sequences with the corresponding rice seedling leaf-age images. The final multimodal dataset comprised both image features and environmental features. The environmental features encompassed accumulated temperature, daily maximum, minimum, and average temperatures, relative air humidity, and soil moisture levels. The integration of these environmental data into the dataset has been shown to facilitate the recognition of rice leaf-age stages, whilst concurrently enabling the M-Lresnet50 model to more accurately characterize rice growth status under multimodal inputs. This, in turn, has been demonstrated to enhance the model’s classification accuracy and robustness.

### 2.2 Dataset configuration

#### 2.2.1 Image dataset configuration

**Figure 3:**
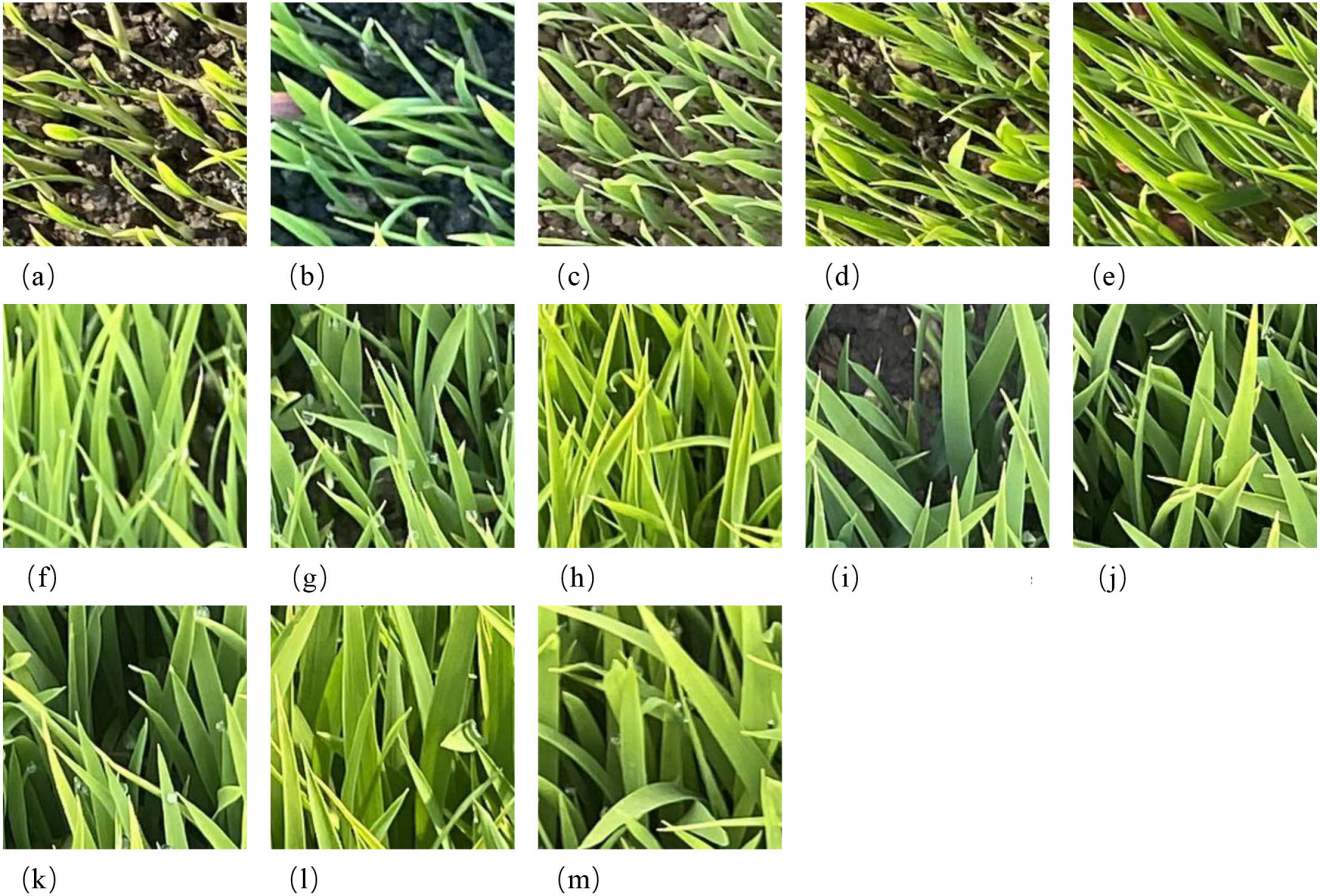
Rice images at different seedling stages. **(a)** 1.1 leaf stage, **(b)** 1.3 leaf stage, **(c)** 1.5 leaf stage, **(d)** 1.7 leaf stage, **(e)** 1.9 leaf stage, **(f)** 2.1 leaf stage, **(g)** 2.3 leaf stage, **(h)** 2.5 leaf stage, **(i)** 2.7 leaf stage, **(j)** 2.9 leaf stage, **(k)** 3.1 leaf stage, **(l)** 3.3 leaf stage, **(m)** 3.5 leaf stage.

The image dataset under consideration consists of rice seedling images collected from different growth stages between April and May 2024. From the array of collected images, a selection of 13 was made to act as representatives of the various seedling leaf-age stages, as illustrated in **Figure 3**. The total number of images captured was 13,740. Following a thorough cleaning and annotation process, samples that were blurry, heavily occluded, or poorly lit were eliminated. Subsequently, each original image was cropped into multiple 224×224 patches to match the model input size. In order to ensure balanced data distribution across different seedling stages and to avoid temporal bias during model training, equal sampling was conducted for each stage. A total of three agronomy experts manually reviewed and corrected the annotations with a view to ensuring accurate classification of the seedling stages. The final curated dataset comprised 144,144 verified images. For the purposes of experimentation, the dataset was randomly divided into training and test sets at a ratio of 8:1, with the remaining portion serving as the validation set. Ensure that no region from the original image appears in all three sets simultaneously: the training set, the validation set and the test set. Subsequent experiments in this study were all based on this dataset. The number of images for each rice seedling stage is provided in **Table 1**.

**Table 1:** Number of images at different rice seedling stages.

Rice seedling age	Date	Training set per image	Test set per image	Val set per image
1.1	4.16	8870	1108	1110
1.3	4.17	8870	1108	1110
1.5	4.18	8870	1108	1110
1.7	4.19	8870	1108	1110
1.9	4.23	8870	1108	1110
2.1	4.26	8870	1108	1110
2.3	4.28	8870	1108	1110
2.5	5.01	8870	1108	1110
2.7	5.04	8870	1108	1110
2.9	5.06	8870	1108	1110
3.1	5.09	8870	1108	1110
3.3	5.11	8870	1108	1110
3.5	5.13	8870	1108	1110

#### 2.2.2 Time-series environmental dataset

In order to facilitate the classification of rice seedlings at different leaf-age growth stages, the collected data were categorized into labels corresponding to key developmental stages: The following leaf stages are recognized: 1.1-leaf, 1.3-leaf, 1.5-leaf, 1.7-leaf, 1.9-leaf, 2.1-leaf, 2.3-leaf, 2.5-leaf, 2.7-leaf, 2.9-leaf, 3.1-leaf, 3.3-leaf, and 3.5-leaf. In order to effectively integrate rice leaf-age image data with environmental factor data, strict temporal synchronization between the two data types was performed. The acquisition of images and the monitoring of the environment were recorded using a unified timestamp. The collection of environmental data was conducted on a continuous basis by sensors, with readings being obtained at one-minute intervals. The data set encompassed nine variables, including temperature, air humidity and soil moisture. The complete set of variables is detailed in **Table 2**. Concurrently, rice leaf images were captured on a daily basis, with each image accompanied by a precise timestamp.

**Table 2:** Environmental data of rice seedling age.

Rice seedling age	Date	Maximum temperature	Minimum temperature	Average temperature	Accumulated temperature	Effective accumulated temperature	Environmental temperature	Environmental humidity	Soil temperature	Soil humidity
1.1	2024/04/16	27.4	1.4	14.4	121.3	41.3	20.9	37.4	13.4	36.8
1.3	2024/04/17	32.5	-0.3	16.1	135.7	45.7	13.4	65.7	13.7	36.7
1.5	2024/04/18	24.4	1	12.7	151.8	51.8	13.6	65.8	10.9	36.1
1.7	2024/04/19	25.8	2.1	13.95	164.5	54.5	25.3	64.1	11.3	41.4
1.9	2024/04/23	24	6.6	15.3	217.25	116.75	23.2	43.8	16.1	30
2.1	2024/04/26	17.5	4	15.75	263.1	132.6	14.3	96.7	13.1	23.8
2.3	2024/04/28	27.6	5.2	16.4	296	145.5	23	64.9	14.1	22.8
2.5	2024/05/01	33.9	7.5	20.7	353.6	173.1	33.2	43.8	21.7	24.9
2.7	2024/05/04	29.5	5.7	17.6	406.4	195.9	12.8	94.2	16.5	26.7
2.9	2024/05/06	30.2	2.3	16.25	439.75	209.25	22.8	37.4	18.7	25.9
3.1	2024/05/09	27.9	10.1	19	496.3	235.8	23.7	60.7	15.9	24.8
3.3	2024/05/11	34.4	5	19.7	533.1	252.6	32.8	41.8	19.2	26.2
3.5	2024/05/13	28.9	12.9	20.9	577.35	276.85	19.3	74	16.4	25.4

During the process of data alignment, the environmental data point that corresponded to the image acquisition timestamp was retrieved. In instances where the timestamp occurred between two environmental sampling points, linear interpolation was employed to calculate the environmental features for that particular moment. In the case of cumulative variables, such as accumulated temperature, the sum over a preceding time window is calculated and assigned to the corresponding image. In the context of fluctuating variables, such as temperature and humidity, the utilization of time-window averaging or extreme values has been employed to mitigate noise. In this process, each image was paired with a fixed-length environmental feature sequence, which served as input to the LSTM module. The integration of the time-series environmental dataset with the structured image dataset resulted in the establishment of a comprehensive multimodal dataset.

#### 2.2.3 Generalization testing dataset configuration

##### 2.2.3.1 Temporal generalization dataset

In order to assess the model’s capacity for adaptation to diverse growing seasons, images of rice seedling of the same varieties were obtained in 2025 at the same experimental location. The dataset under consideration encompassed all 13 leaf-age stages. The same equipment, fixed-point layout, and image capture protocols were utilized to ensure consistency, with the exception of the difference in year. Initially, a total of 6,840 images were collected; following the filtration and cropping process, 14,404 images remained, with 1,108 images per leaf-age stage. The dataset under consideration was meticulously designed to evaluate the model’s robustness to interannual climate variation.

##### 2.2.3.2 Spatial generalization dataset

In order to assess the model’s performance across different geographical environments and greenhouse management practices, the present study collaborated with the Wuchang Agricultural IoT Service Center to obtain rice seedling images from experimental greenhouses in Wuchang, Harbin, Heilongjiang Province. The dataset under consideration focused on the locally cultivated variety “Daohuaxiang No. 2”. The data were collected between April and May 2024, covering the same 13 leaf-age stages, and followed the same image acquisition protocol as the main study. Initially, 6,840 images were captured; subsequent filtration and cropping resulted in 14,404 images, with 1,108 images per stage. The dataset was utilized to evaluate the model’s spatial transferability.

##### 2.2.3.3 Cross-cultivar generalization dataset

In order to evaluate the model’s ability to recognize unseen rice varieties, a completely new cultivar, “Qijing No.10,” which was not present in the primary dataset, was introduced. The images were collected between April and May 2024 from the Beidahuang Xu Yirong High-Tech Rice Demonstration Park in Hulin, Jixi, Heilongjiang Province. The annotation of the images was conducted in strict accordance with the five-point leaf-age evaluation method. The dataset was designed for a “leave-one-variety-out” validation strategy, which was employed to rigorously test the transferability of the learned features. Initially, 6,840 images were collected, and following the filtration and cropping processes, 14,404 images were finalized, with 1,108 images per stage.

### 2.3 Proposed Lresnet50 model

Lresnet50 improves upon the Resnet50 backbone by integrating three key modules that enhance its ability to recognize rice seedlings in complex field conditions. The Row-Prior Strip Attention module uses row-planting information to focus on seedling structures and suppress background noise through decomposed, strip-wise attention. The Feature Pyramid Network combines multi-scale features to improve the perception of variations in leaf size across growth stages. Finally, the Dynamic Channel Pruning module adjusts channel weights in real time based on the input, significantly reducing computational costs while maintaining accuracy. Together, these modules provide the model with enhanced discriminative power, multi-scale adaptability and deployment efficiency.

#### 2.3.1 Row-prior strip-attention design

Rice is a conventionally cultivated crop, characterized by the elongation of its leaves along the ridge. These leaves manifest subtle variations in length, posture, and orientation across different seedling stages. Conventional spatial attention mechanisms have been shown to enhance salient regions; however, their two-dimensional convolutions do not explicitly incorporate the prior of row planting. This results in difficulties in highlighting strip-like leaf structures and renders the model susceptible to interference from soil, weeds and shadows in complex field backgrounds. In order to address this issue, the RPS module is proposed, which is embedded after the C3 and C4 layers of the Resnet50 backbone in order to leverage spatial priors of crop rows during feature extraction.

The RPS module decomposes 2D spatial attention into two independent 1D strip-attention branches: the horizontal branch models the long leaf structures along the row direction, while the vertical branch captures texture variations across rows. In the context of an input feature map F, the initial procedure entails the implementation of horizontal global strip pooling, a process that results in the compression of the entire row into a channel vector as shown in Equation (1):

(1) $$F_{h}(i,c)=\frac{1}{W}\sum_{j=1}^{W}F(i,j,c)$$

where F(i,j,c) is the response of channel c at position (i,j), and W is the feature map width.

This operation preserves spatial resolution along rows while discarding column-wise positional information, focusing on leaf distribution patterns along the row. Similarly, vertical global strip pooling is applied as shown in Equation (2):

(2) $$F_{v}(j,c)=\frac{1}{H}\sum_{i=1}^{H}F(i,j,c)$$

where *H* is the feature map height. This captures column-wise spatial structure, characterizing cross-row morphological variations and weed interference patterns.

To efficiently capture local textures within strips, $F_{h}$ and $F_{v}$ are processed with depthwise-separable convolutions. The depthwise convolution performs 1D convolutions within channels to enhance direction-specific textures, while the pointwise convolution mixes channels to account for inter-channel correlations. Compared to standard 2D convolutions, this significantly reduces parameters and computation, suitable for lightweight field deployment.

The processed $F_{h}$​ and $F_{v}$ are mapped to the same channel dimension and fused via addition to generate direction-aware attention weights *M* as shown in Equation (3):

(3) $$M=\sigma (W_{h}F_{h}+W_{v}F_{v})$$

where σ is the sigmoid function mapping weights to [0,1]. Finally, the attention map *M* is multiplied element-wise with the original feature map *F* for feature enhancement. This effectively introduces direction-prior modulation to each feature position, highlighting regions consistent with row distribution and suppressing background noise irrelevant to seedling age. By embedding RPS in the C3 and C4 layers, mid-to-high-level semantic features already incorporate row geometry priors, improving model focus on rice plant structure. Since seedling age detection relies on fine-grained differences in leaf posture, length, and arrangement, this module enhances discriminative power with minimal computational cost, improving robustness under low-light and complex field conditions.

#### 2.3.2 Feature pyramid network

To capture multi-scale features and improve recognition of small targets, a FPN is incorporated on top of the backbone. FPN uses a top-down pathway with lateral connections to propagate high-level semantic features to lower layers, enhancing semantic representation while maintaining spatial resolution.

Let the backbone output multi-scale feature maps $\left\{C_{2},C_{3},C_{4},C_{5}\right\}$, where $C_{l}$​ denotes features from stage l. Low-level features have high resolution but weak semantics, while high-level features are semantically rich but low in resolution.

First, the top-level feature $C_{5}$​ is reduced in dimension via a 1×1 convolution as shown in Equation (4):

(4) $$P_{5}=W_{5}*C_{5},$$

where $W_{5}$​ is the convolution kernel. $P_{5}$ is then upsampled and added to the aligned lower-level feature.

For each feature layer $C_{l}$, a 1×1 convolution is similarly applied for channel alignment. The upsampled higher-level feature $P_{l+1}$ is then added to it, forming a new fused feature as shown in Equation (5):

(5) $$P_{l}=W_{l}*C_{l}+\text{Up}(P_{l+1})$$

Where up denotes the upsampling operation. This process continues for all levels. Each fused feature $P_{l}$ is further smoothed with a 3×3 convolution to reduce aliasing as shown in Equation (6):

(6) $$P_{l}^{'}={\text{Conv}}_{3\times 3}(P_{l})$$

The resulting pyramid $\left\{P2^{\prime },P3^{\prime },P4^{\prime },P5^{\prime }\right\}$ contains rich semantic information and maintains high spatial resolution, enabling detection and recognition across multiple scales.

#### 2.3.3 Dynamic channel pruning

Deep CNNs often have redundant channels, which increase computation during inference. To address this, the DCP module adaptively weights channels to suppress redundant features and enhance important ones. Unlike static pruning, DCP adjusts channel importance based on the input, improving efficiency without sacrificing accuracy.

For input feature $X\in {\mathbb{R}}^{B\times C\times H\times W}$, where *B* is batch size, *C* is the number of channels, and H,W are spatial dimensions, global average pooling (GAP) compresses each channel into a scalar as shown in Equation (7):

(7) $$z_{c}=\frac{1}{H\times W}\sum_{i=1}^{H}\sum_{j=1}^{W}X_{c}(i,j),z=[z_{1},z_{2},\ldots ,z_{C}]\in {\mathbb{R}}^{C}$$

The obtained channel descriptor vector *z* represents the global response of each channel. Subsequently, a two-layer fully connected bottleneck network (bottleneck MLP) is utilized to model the dependencies between channels as shown in Equation (8):

(8) $$s=\sigma (W_{2}\delta (W_{1}z))$$

where *r* is the channel compression ratio, δ is ReLU, and σ is sigmoid. The weight vector is obtained after this mapping as shown in Equation (9):

(9) $$s=[s_{1},s_{2},\ldots ,s_{C}],s_{c}\in (0,1)$$

**Figure 4:**
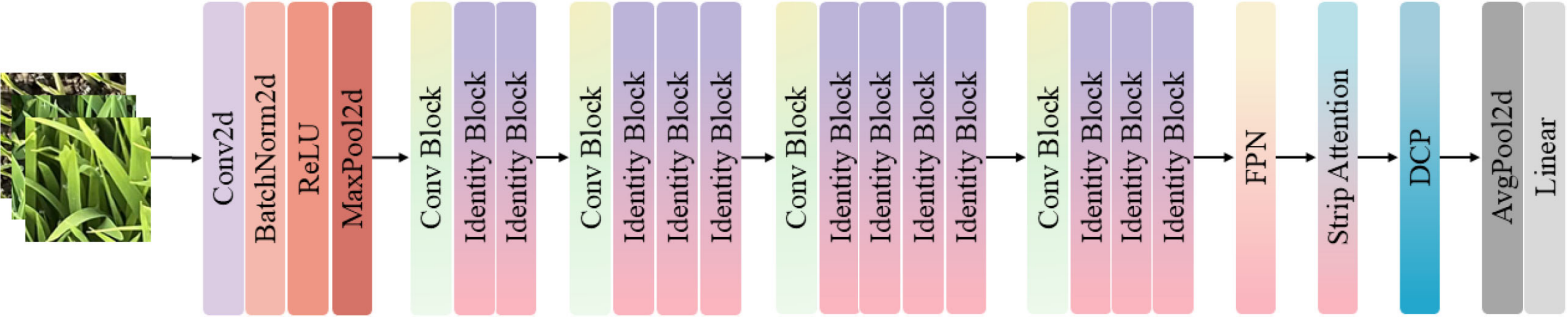
Architecture of the Lresnet50 model.

Finally, the DCP applies these weights channel-wise to the original feature maps to achieve dynamic channel selection as shown in Equation (10):

(10) $$X_{c}^{'}=s_{c}\cdot X_{c},c=1,2,\ldots ,C$$

where $X_{c}^{"}$ denotes the weighted output channel. If $s_{c}$ approaches 0, the channel is “suppressed” during the current inference process, equivalent to performing soft pruning; whereas if $s_{c}$ approaches 1, the channel is fully retained and its feature transmission is enhanced. The structure diagram of the Lresnet50 model is shown in **Figure 4**.

### 2.4 Proposed M-Lresnet50 model

**Figure 5:**
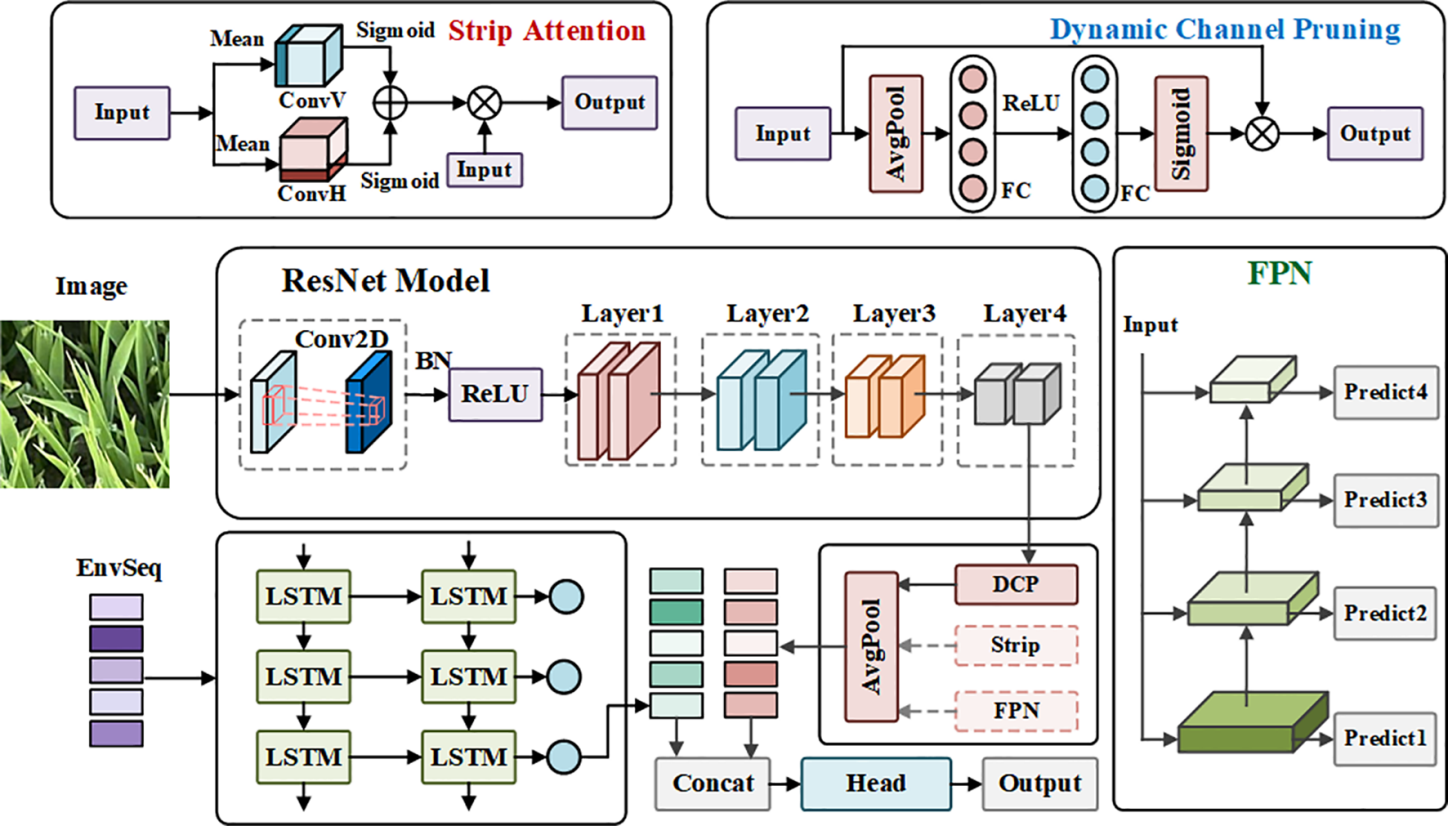
Architecture of the M-Lresnet50 model.

The M-Lresnet50 network is comprised of two core modules: an image feature extraction module and an environmental feature extraction module. Multi-modal fusion is implemented at the feature fusion layer, as illustrated in **Figure 5**.

The model utilizes the enhanced Resnet50 backbone, which has been augmented with three distinct mechanisms, for the purpose of image feature extraction. FPN, RPS, and DCP. The modified Resnet50 model first integrates multi-scale features via a deep convolutional neural network (CNN) and concatenates them at a unified scale. Subsequently, a pooling operation is employed to yield a 1024-dimensional image feature vector, which is then subjected to further processing through global average pooling to generate the final image feature representation. In the context of environmental feature extraction, a two-layer stacked LSTM network is utilized, with each layer comprising 64 hidden units. The input is constituted by a temporal sequence of length 2, comprising 9 environmental feature dimensions. The output of the model employs the hidden state at the final time step as a 64-dimensional environmental feature representation. In order to mitigate the issue of overfitting, a dropout rate of 0.3 is applied.

During the multimodal feature fusion process, the image features extracted by the CNN and the environmental time-series features encoded by the LSTM are first concatenated along the channel dimension to form a unified joint representation. This concatenation preserves the complementary strengths of both modalities without losing their original information. The fused feature vector is then processed through a two-layer fully connected network: the first layer reduces its dimension by half using a ReLU activation to enhance non-linear representation ability, while the second layer performs a linear mapping to output the final prediction across the 13 rice seedling age classes. By integrating spatial visual cues with sequential environmental patterns, M-Lresnet50 effectively aligns cross-modal information and enhances feature discriminability. This design enables complementary multimodal fusion, which has been shown to significantly improve the robustness and accuracy of rice seedling age recognition under complex field conditions.

## 3 Results and analysis

### 3.1 Environment and parameter configuration

In this study, the training of the Lresnet50 model is heavily reliant on deep learning tasks and requires GPU acceleration to meet computational demands. The primary hardware configuration employed in the experimental setup consists of an Intel Core i7-14650HX processor operating at a frequency of 2.20 GHz, an NVIDIA GeForce GTX 4060 GPU, and 16 GB of RAM. The software environment is based on the Windows 11 operating system, with CUDA 11.3 and Python 3.12 configured via Anaconda. All programming and experiments were carried out using PyCharm 2024.1.2 as the integrated development environment. As illustrated in **Table 3**, a comprehensive synopsis of the hardware and software configurations employed in the experimental setup is provided. This experimental platform offered strong support for large-scale data processing and the computational demands of complex neural networks. The employment of a dedicated GPU has been demonstrated to result in a substantial enhancement in the training and inference speed of the model. This development has enabled accelerated iterations and expanded experimental exploration, thereby facilitating more efficient progress in the research domain.

**Table 3:** Experimental environment.

Category	Environment parameter
Operating system	Windows 11–64 bits
Development tool	PyCharm
CPU	Intel Core i7-14650HX 2.20 GHz
GPU	NVIDIA GeForce GTX 4060
Deep learning framework	Pytorch
Scripting language	Python 3.12
RAM	16.0 GB

In order to optimize the model, the SGD algorithm was employed as the optimizer. The batch size was set to 32, the learning rate was set to 0.01, the momentum parameter was set to 0.9, and four data loading threads were enabled. Furthermore, a cosine annealing strategy was employed to enable the dynamic adjustment of the learning rate throughout the training process. The hyperparameters have been selected and tuned with the objective of enhancing the model’s ability to efficiently learn from the training data while improving its generalization performance on unseen samples. Consequently, the optimization measures effectively enhanced the accuracy of Lresnet50 in rice seedling stage classification tasks.

### 3.2 Evaluation metrics

In order to provide a comprehensive evaluation of the model’s performance in identifying rice seedlings, a range of evaluation metrics were employed in this study:

Confusion Matrix: The confusion matrix (Sokolova and Lapalme, 2009) evaluates the accuracy of classification models by comparing the correspondence between actual and predicted classes. It consists of four key elements: True Positive (TP), False Positive (FP), True Negative (TN), and False Negative (FN). These indicators allow for detailed analysis of model performance, especially in scenarios with class imbalance.

Accuracy: Accuracy measures the proportion of correctly classified samples among all samples and is calculated using Equation (11):

(11) $$Accuracy=\frac{TP+TN}{TP+FN+FP+TN}$$

Precision: Precision reflects the proportion of correctly predicted positive samples among all samples predicted as positive. It is calculated using Equation (12):

(12) $$Precision=\frac{TP}{TP+FP}$$

Recall: Recall indicates the proportion of actual positive samples that were correctly identified by the model. It is calculated using Equation (13):

(13) $$Recall=\frac{TP}{TP+FN}$$

F1-Score: The F1-score is the harmonic mean of precision and recall, providing a balanced evaluation by considering both false positives and false negatives. It is calculated using Equation (14):

(14) $$F1=\frac{2\cdot Precision\cdot Recall}{Precision+Recall}$$

Params: This metric reflects the total number of trainable parameters in the rice seedling stage detection model.

GFLOPs (Floating Point Operations): GFLOPs represent the computational cost of the model, which serves as an indicator of model complexity.

### 3.3 Lresnet50 ablation test

**Figure 6:**
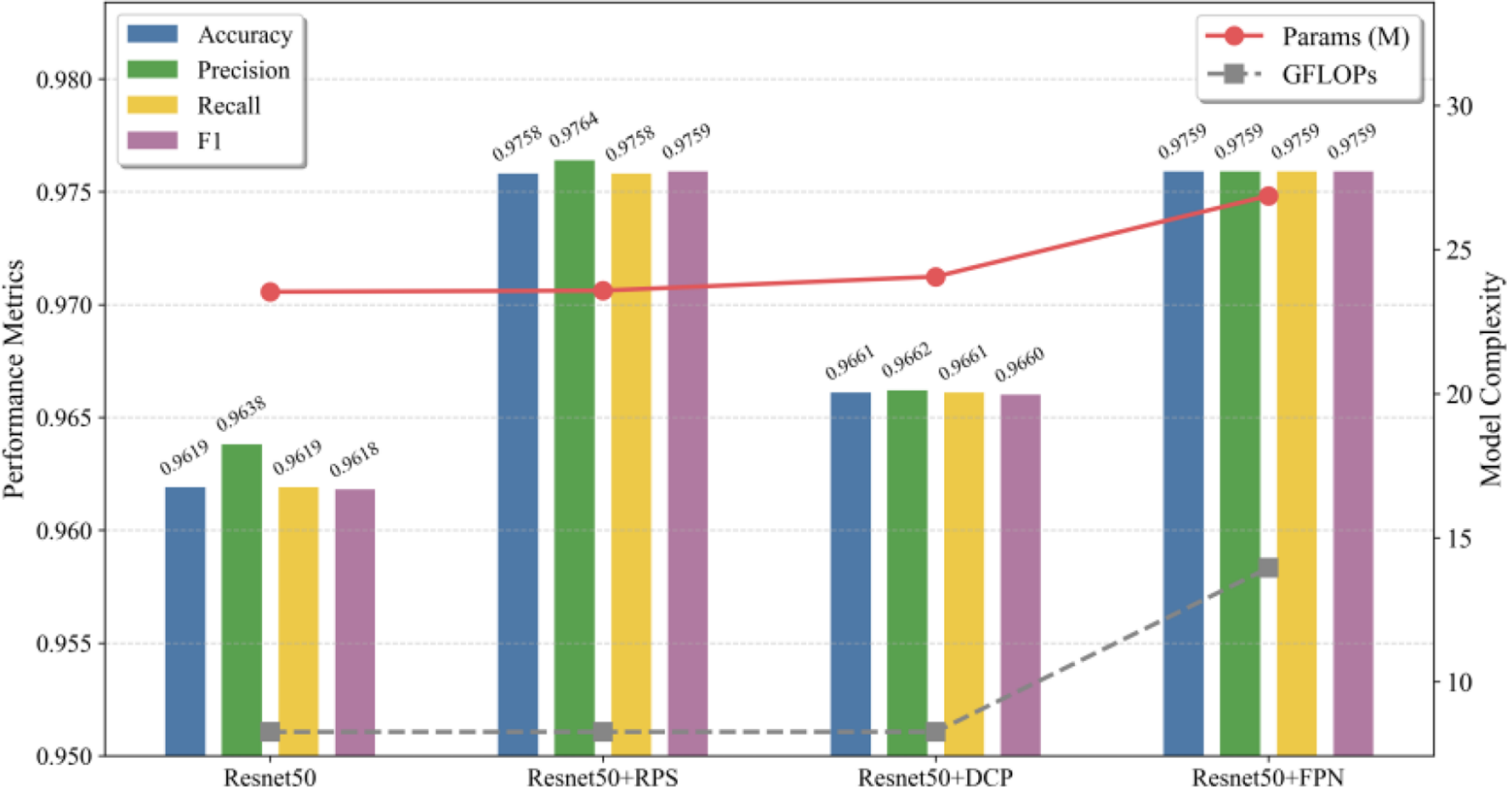
Ablation experiment.

The results of the ablation experiment are presented in **Table 4** and **Figure 6**. A systematic analysis of these results clearly evaluates the contributions of each improved module to the baseline model’s performance. The findings demonstrate that all introduced modules enhance the model’s performance to varying degrees, thus demonstrating their effectiveness in rice seedling age recognition tasks.

**Table 4:** Ablation experiment.

Models	Accuracy	Precision	Recall	F1	Params	GFLOPs
Resnet50	0.9619	0.9638	0.9619	0.9618	23.535	8.263
Resnet 50+RPS	0.9758	0.9764	0.9758	0.9759	23.576	8.264
Resnet 50+DCP	0.9661	0.9662	0.9661	0.9660	24.059	8.265
Resnet 50+FPN	0.9759	0.9763	0.9759	0.9759	26.866	13.954

Firstly, the RPS module demonstrated remarkable performance enhancement capabilities. A minimal increase of only 0.041M parameters and 0.001 GFLOPs was observed, yet this nonetheless resulted in a significant enhancement of model accuracy from 0.9619 to 0.9758, achieving an absolute gain of 1.39 percentage points. Of particular significance was the fact that these improvements were consistent and balanced across four key metrics. The metrics of accuracy, precision, recall, and the F1-score all demonstrated a high level of precision, with values of 0.9758, 0.9764, 0.9758, and 0.9759, respectively. This comprehensive performance enhancement indicates that by incorporating row-crop spatial prior knowledge, the RPS module strengthens the model’s perception of the distinctive distribution patterns of rice leaves. Consequently, it enables the model to better focus on discriminative regions related to seedling age, achieving superior performance with extremely high cost-effectiveness. In comparison, the FPN module achieved a comparable level of performance, attaining an accuracy of 0.9759. However, this enhancement was accompanied by a substantially elevated computational expense, necessitating the incorporation of 3.331 million additional parameters and 5.691 GFLOPs. The multi-scale feature fusion technique has been shown to be an effective method of capturing morphological changes in rice seedlings at different growth stages. This approach has been found to be particularly advantageous when dealing with objects that exhibit large-scale variations. This capacity is of particular significance for the recognition of rice seedling age, given that divergent growth stages manifest substantial phenotypic variations. The DCP module, on the other hand, emphasized a distinct optimization focus. Although the improvement in accuracy was relatively limited to 0.42 percentage points, the module only increased 0.524M parameters and 0.002 GFLOPs in terms of the number of parameters and computational load. This renders DCP especially well-suited for implementation in environments where resources are limited, thus providing a pragmatic solution for the development of lightweight models. The findings of the present study provide a robust validation of the efficacy of the constituent modules, thus offering a scientific foundation for the selection of model components in a range of application scenarios.

**Figure 7:**
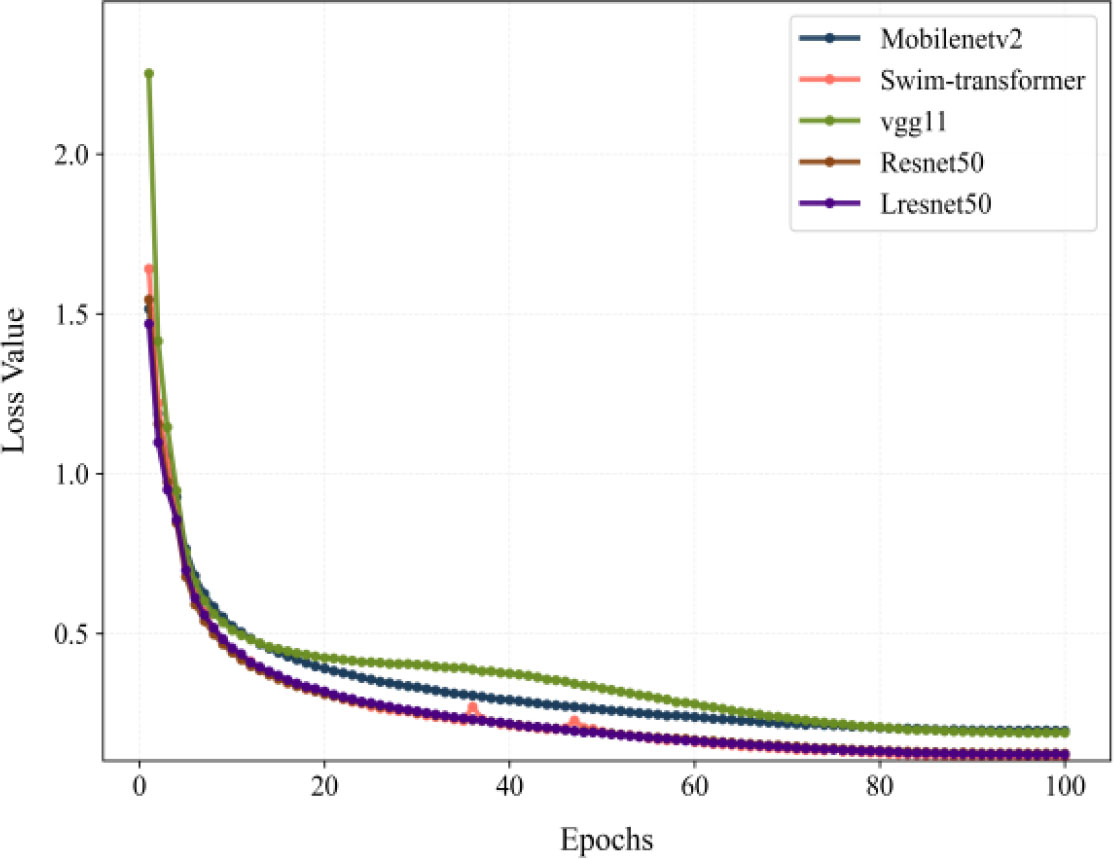
Loss curves of different models.

In order to further assess the capability of the model, a comparison of different architectures was made. A comparison was made between standard convolutional neural networks and the purpose-built Lresnet50 model, the latter of which was designed for the purpose of monitoring the age of rice seedlings. The comparison comprised Mobilenetv2, vgg11, Swin-transformer, Resnet50, and Lresnet50, all of which are well-established in computer vision research with strong generalization capabilities. As illustrated by the loss curves in **Figure 7**, all models demonstrated a rapid decline in loss during the initial 10 epochs, subsequently converging gradually, with discernible performance variations. Swin-transformer, Lresnet50 and Resnet50 achieved the lowest losses and fastest convergence, indicating strong optimizability and stability. Conversely, vgg11 consistently demonstrated the highest loss and the slowest convergence rate, indicative of its comparatively weaker optimization performance. In summary, the Lresnet50 allows for superior convergence efficiency in comparison with traditional vgg architectures.

### 3.4 Lresnet50 comparative test

**Figure 8:**
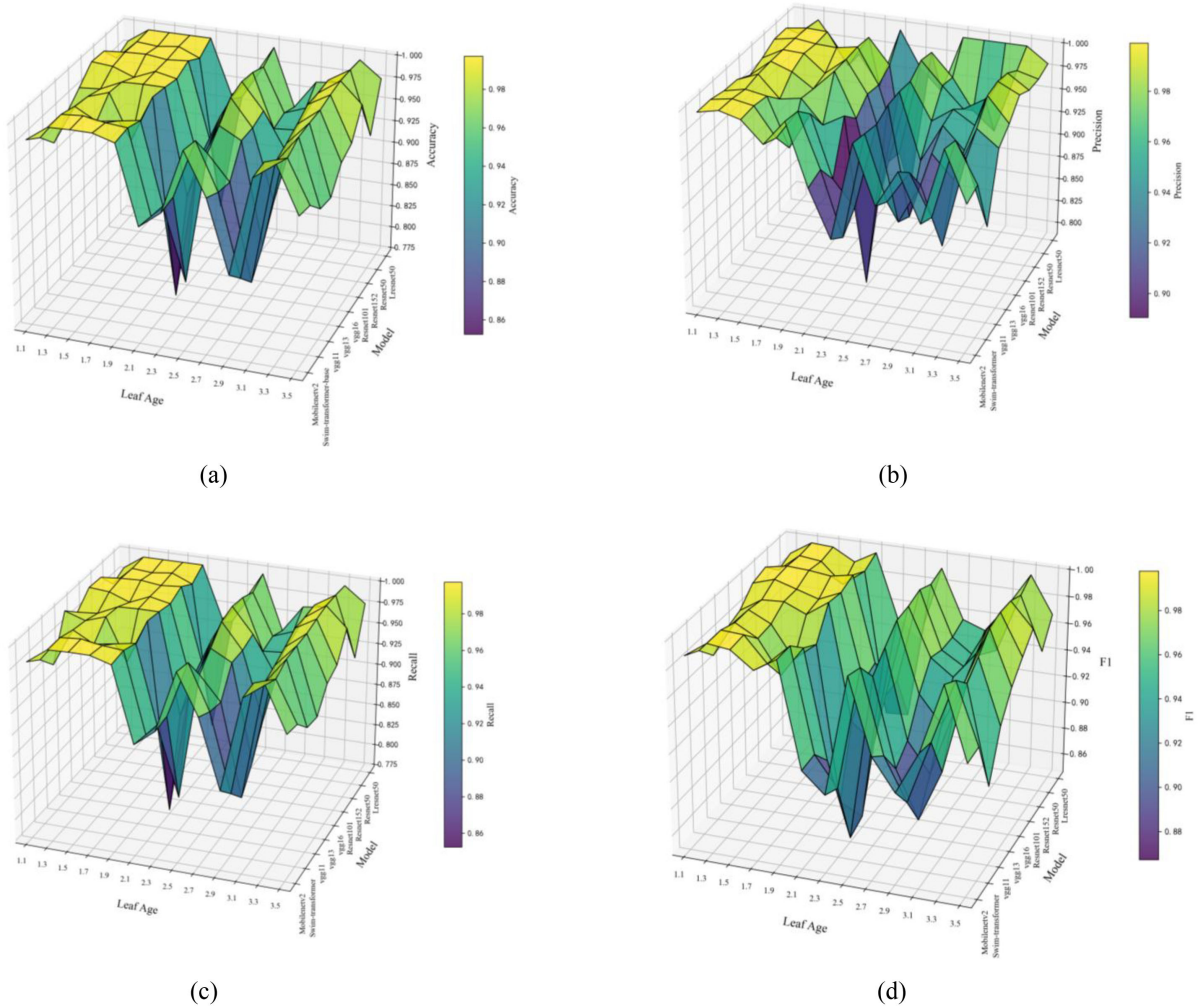
Comparative test. **(a)** Accuracy of different models across various seedling stages, **(b)** Precision of different models across various seedling stages, **(c)** Recall of different models across various seedling stages, **(d)** F1 of different models across various seedling stages.

A systematic comparative analysis was conducted between typical convolutional neural networks and the proposed Lresnet50 model. The present study elected to utilize a number of widely adopted models as baselines, including Mobilenetv2 (Chen et al., 2018), Swin-transformer (Liu et al., 2021), and the Resnet (He et al., 2015) family. The validation of these models across various domains has demonstrated their excellent performance. The results obtained in the rice seedling age recognition task are presented in **Tables 5**–**9** and **Figure 8**.

**Table 5:** Accuracy of different models across various seedling stages.

Rice seedling age	Mobilenetv2	Swim-transformer	vgg11	vgg13	vgg16	Resnet101	Resnet152	Resnet50	Lresnet50
1.1	0.979	0.967	0.947	0.966	0.981	0.997	0.996	0.975	0.982
1.3	0.996	0.996	0.991	0.988	0.983	0.995	0.998	0.997	0.998
1.5	0.997	0.998	0.978	0.996	0.994	0.986	0.999	0.995	0.998
1.7	0.997	0.994	0.982	0.947	0.995	0.996	0.996	0.998	0.998
1.9	0.990	0.992	0.986	0.998	0.995	0.991	0.998	0.988	0.998
2.1	0.903	0.899	0.893	0.777	0.876	0.902	0.912	0.818	0.936
2.3	0.922	0.924	0.813	0.927	0.937	0.955	0.962	0.950	0.958
2.5	0.993	0.989	0.961	0.977	0.994	0.998	0.995	0.988	0.997
2.7	0.946	0.937	0.933	0.964	0.964	0.969	0.935	0.939	0.932
2.9	0.866	0.846	0.825	0.851	0.885	0.929	0.937	0.948	0.964
3.1	0.975	0.977	0.962	0.968	0.975	0.983	0.982	0.983	0967
3.3	0.993	0.985	0.993	0.994	0.994	0.997	0.999	0.998	0.996
3.5	0.942	0.938	0.919	0.916	0.940	0.947	0.976	0.927	0.978

**Table 6:** Precision of different models across various seedling stages.

Rice seedling age	Mobilenetv2	Swim-transformer	vgg11	vgg13	vgg16	Resnet101	Resnet152	Resnet50	Lresnet50
1.1	0.995	0.995	0.991	0.990	0.982	0.988	0.996	0.996	0.978
1.3	1.000	0.999	0.996	0.999	1.000	1.000	1.000	0.999	0.996
1.5	0.994	0.995	0.992	0.976	0.992	0.999	0.997	0.998	0967
1.7	0.972	0.965	0.941	0.980	0.977	0.981	0.996	0.964	0.964
1.9	0.999	0.996	0.980	0.955	0.999	0.999	1.000	0.998	0.932
2.1	0.934	0.939	0.915	0.968	0.952	0.965	0.962	0.971	0.997
2.3	0.884	0.871	0.888	0.791	0.861	0.895	0.891	0.835	0.958
2.5	0.987	0.983	0.979	0.986	0.987	0.985	0.995	0.991	0.936
2.7	0.926	0.916	0.880	0.871	0.917	0.946	0.971	0.967	0.998
2.9	0.965	0.950	0.923	0.942	0.958	0.962	0.949	0.934	0.998
3.1	0.915	0.907	0.862	0.918	0.935	0.948	0.963	0.915	0.998
3.3	0.995	0.990	0.957	0.975	0.996	0.998	0.994	0.984	0.998
3.5	0.943	0.948	0.890	0.958	0.970	0.987	0.979	0.978	0.982

**Table 7:** Recall of different models across various seedling stages.

Rice seedling age	Mobilenetv2	Swim-transformer	vgg11	vgg13	vgg16	Resnet101	Resnet152	Resnet50	Lresnet50
1.1	0.979	0.967	0.947	0.996	0.981	0.997	0.996	0.975	0.982
1.3	0.996	0.996	0.991	0.988	0.983	0.995	0.998	0.997	0.998
1.5	0.997	0.998	0.978	0.996	0.994	0.986	0.999	0.995	0.998
1.7	0.997	0.994	0.982	0.947	0.995	0.996	0.996	0.998	0.998
1.9	0.990	0.992	0.986	0.998	0.995	0.991	0.998	0.988	0.998
2.1	0.903	0.899	0.893	0.777	0.876	0.902	0.912	0.818	0.936
2.3	0.922	0.924	0.813	0.927	0.937	0.955	0.962	0.950	0.958
2.5	0.993	0.989	0.961	0.977	0.994	0.998	0.995	0.988	0.997
2.7	0.946	0.937	0.933	0.964	0.964	0.969	0.935	0.939	0.932
2.9	0.866	0.846	0.825	0.851	0.885	0.929	0.937	0.948	0.964
3.1	0.975	0.977	0.962	0.968	0.975	0.983	0.982	0.983	0.967
3.3	0.993	0.985	0.993	0.994	0.994	0.997	0.999	0.998	0.996
3.5	0.942	0.938	0.919	0.916	0.940	0.947	0.976	0.927	0.978

**Table 8:** F1 of different models across various seedling stages.

Rice seedling age	Mobilenetv2	Swim-transformer	vgg11	vgg13	vgg16	Resnet101	Resnet152	Resnet50	Lresnet50
1.1	0.987	0.981	0.968	0.978	0.982	0.993	0.996	0.985	0.989
1.3	0.998	0.998	0.994	0.994	0.992	0.998	0.999	0.998	0.999
1.5	0.996	0.997	0.985	0.986	0.993	0.993	0.998	0.996	0.998
1.7	0.984	0.979	0.961	0.963	0.986	0.988	0.996	0.981	0.989
1.9	0.994	0.994	0.983	0.976	0.997	0.995	0.999	0.993	0.998
2.1	0.918	0.919	0.904	0.862	0.912	0.932	0.936	0.888	0.951
2.3	0.903	0.897	0.849	0.854	0.898	0.924	0.925	0.889	0.937
2.5	0.990	0.986	0.970	0.981	0.991	0.992	0.995	0.990	0.993
2.7	0.936	0.926	0.906	0.916	0.940	0.957	0.953	0.953	0.957
2.9	0.913	0.895	0.871	0.894	0.920	0.945	0.943	0.941	0.951
3.1	0.944	0.941	0.909	0.942	0.955	0.965	0.973	0.948	0.972
3.3	0.994	0.987	0.975	0.984	0.995	0.997	0.996	0.991	0.997
3.5	0.942	0.943	0.904	0.937	0.955	0.967	0.978	0.952	0.970

**Table 9:** Params and GFLOPs of different models.

Indicator	Mobilenetv2	Swim-transformer	vgg11	vgg13	vgg16	Resnet101	Resnet152	Resnet50	Lresnet50
Params	2.241	86.693	128.820	129.004	134.314	45.527	58.170	23.535	27.017
GFLOPs	652.447	30.338	15.210	22.609	30.933	15.729	23.204	8.263	13.962

The findings indicate that Lresnet50 demonstrates superior performance in comparison to all baseline models across the designated performance metrics. On average, for the 13 rice seedling growth stages, the Accuracy and Precision of Resnet50 both reached 0.977, a figure significantly higher than that of Resnet152 and Resnet50. In a stage-by-stage performance analysis, Lresnet50 demonstrated the highest levels of Accuracy and Precision in approximately 50% of the stages, and was on par with the best models in multiple stages. This finding reflects the model’s stable, high-precision recognition capability.

It is worth noting that the transitional growth stages are the most challenging to distinguish, due to subtle morphological differences, significant leaf overlap, and strong background interference. The experimental results obtained demonstrate that Lresnet50 has achieved substantial improvements in these stages. At Stage 2.1, Accuracy attained 0.936, representing an 11.8 percentage point increase over Resnet50, while Precision exhibited a 2.6 points improvement to 0.997. At the exceedingly challenging Stage 2.3, Precision attained 0.958, a figure 12.3 points higher than that of Resnet50, thus signifying the most substantial enhancement observed across the entire sequence of stages. In a similar vein, at Stages 1.9, 2.9, and 3.5, Accuracy saw improvements of 1.0, 1.6, and 5.1 points, respectively, over the baseline models. The findings demonstrate that Lresnet50 is highly effective in differentiating visually similar transitional stages, thereby effectively reducing confusion between adjacent growth phases.

From a cross-stage stability perspective, Lresnet50 demonstrated superior robustness. A comparison of the recall and F1-score metrics reveals that Lresnet50 demonstrated optimal or near-optimal performance in the majority of stages. This observation indicates that Lresnet50 exhibited a consistent high-performance plateau during transitional phases. The ability to adapt to occlusion, background noise, and minor morphological variations is of the utmost importance for uninterrupted monitoring and dynamic recognition in precision agriculture.

With regard to the complexity-performance trade-off, it is evident that Lresnet50 achieved a well-balanced design. While the Lresnet50 model necessitated slightly more parameters and computations, the performance gains were substantial. In contrast, Resnet152 and Swin-transformer exhibited significantly higher complexities without demonstrating superior performance to Lresnet50. This demonstrates that Lresnet50 achieves superior performance within an acceptable computational budget, making it highly deployable and practical, especially in agricultural environments where resources are limited.

In summary, experimental findings provide substantial validation of the advantages of Lresnet50 in the recognition of rice seedling age. The model exhibits superior performance in comparison to baseline models, demonstrating consistent performance above the mean average. Furthermore, it evinces notable enhancements in the challenging transitional stages of the task. Furthermore, it strikes a balance between complexity and efficiency, rendering it suitable for real-world agricultural applications. These fine-grained recognition strengths provide more accurate and timely decision support for precision agriculture, thus highlighting Lresnet50’s potential for widespread use in production settings. Notwithstanding, there are risks of misclassification that persist during transitional stages. To further enhance performance, an improved method combining multimodal data features is proposed, which strengthens the model’s discriminative ability by introducing environmental data outside the image. This strategy provides a more robust framework for precise rice seedling age classification.

### 3.5 Heatmap validation experiment of M-Lresnet50

**Figure 9:**
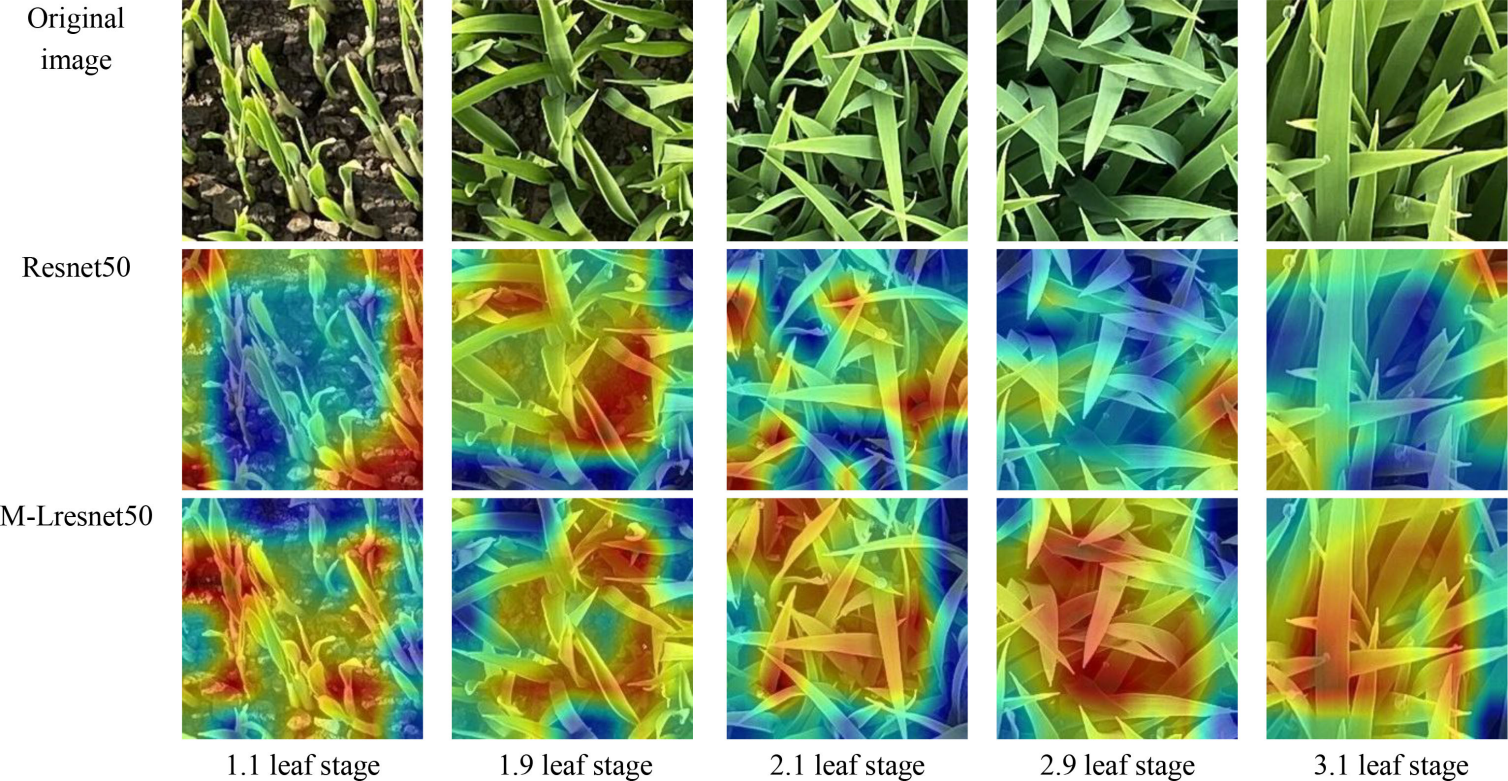
Heat maps of M-Lresnet50 and Resnet50 model at different leaf stages.

To verify the feature extraction capability of the M-Lresnet50 model for rice seedling age recognition, five representative growth stages 1.1, 1.9, 2.1, 2.9 and 3.1 were selected for analysis. Using the Grad-CAM (Selvaraju et al., 2019) visualization method, which generates class-discriminative activation maps by highlighting image regions that contribute most to the model’s predictions, we compared the regions of interest of the original Resnet50 and M-Lresnet50 models, as shown in **Figure 9**. In the Grad-CAM maps, warmer colors indicate regions that contribute more strongly to the model’s prediction, while cooler colors represent less relevant areas, enabling a clear comparison of the discriminative feature focus between Resnet50 and M-Lresnet50.

In the early stages of seedling growth, rice plants are small and significantly affected by background soil interference. The Resnet50 heatmaps revealed scattered attention regions, with red high-response areas appearing in the background soil. This suggests that the model is distracted by non-target regions, resulting in a lack of focus on the rice seedlings. In contrast, M-Lresnet50 produced heatmaps in which the high-response regions were concentrated on the leaves and growth points of the seedling. This demonstrates that M-Lresnet50 effectively suppresses background noise and accurately captures key structural information. Integrating the RPS, DCP and FPN modules enhances the model’s ability to perceive small-scale targets.

As the seedlings grow, their leaves increase in number and begin to overlap significantly. At this stage, the attention regions of Resnet50 appeared fragmented and lacked a comprehensive understanding of the plant structure, often missing important features. In contrast, M-Lresnet50 displayed a more integrated and continuous attention pattern. Heatmaps showed coherent, high-response regions that fully covered the main leaf veins and morphological features. In the late seedling stage, the leaves become densely overlapped and the plant becomes substantially denser. Resnet50 heatmaps showed misjudgements and blurring with weak responses in some leaf areas, indicating difficulty in distinguishing fine differences among dense plants. By contrast, M-Lresnet50 maintained clear, stable attention patterns that covered the main structures of the entire plant cluster, suppressing background interference and shadow effects. This suggests that the environmental data provided temporal growth cues that compensated for ambiguous or occluded visual information, ultimately enhancing the model’s discriminative capability and robustness.

### 3.6 Comparison between M-Lresnet50 and Lresnet50

M-Lresnet50 is a multimodal data classification model that combines features extracted by separate image and environmental feature extraction networks for final classification. The main purpose of this experiment is to verify the effectiveness of multimodal feature fusion. The performance of unimodal and multimodal inputs was compared based on accuracy, precision, recall and F1-score, as shown in **Table 10**, and the results were analyzed in detail.

**Table 10:** Performance comparison between Lresnet50 and M-Lresnet50.

Models	Accuracy	Precision	Recall	F1	Params	GFLOPs
Lresnet50	0.9770	0.9774	0.9770	0.9771	27.017	13.962
M-Lresnet50	0.9833	0.9836	0.9833	0.9833	27.656	13.965

In order to evaluate the effectiveness of multimodal features in rice seedling age recognition, the performance of the unimodal Lresnet50 and the multimodal M-Lresnet50 models were compared on the same test set. As shown in **Table 10**, the overall accuracy of Lresnet50 was 0.9770, whereas M-Lresnet50 increased to 0.9833. Overall precision improved from 0.9774 to 0.9836, an increase of 0.62%. Recall increased by 0.63% to 0.9833, and the overall F1 score increased from 0.9771 to 0.9833. These improvements suggest that incorporating multimodal environmental features can significantly enhance the model’s discriminative capability.

**Figure 10:**
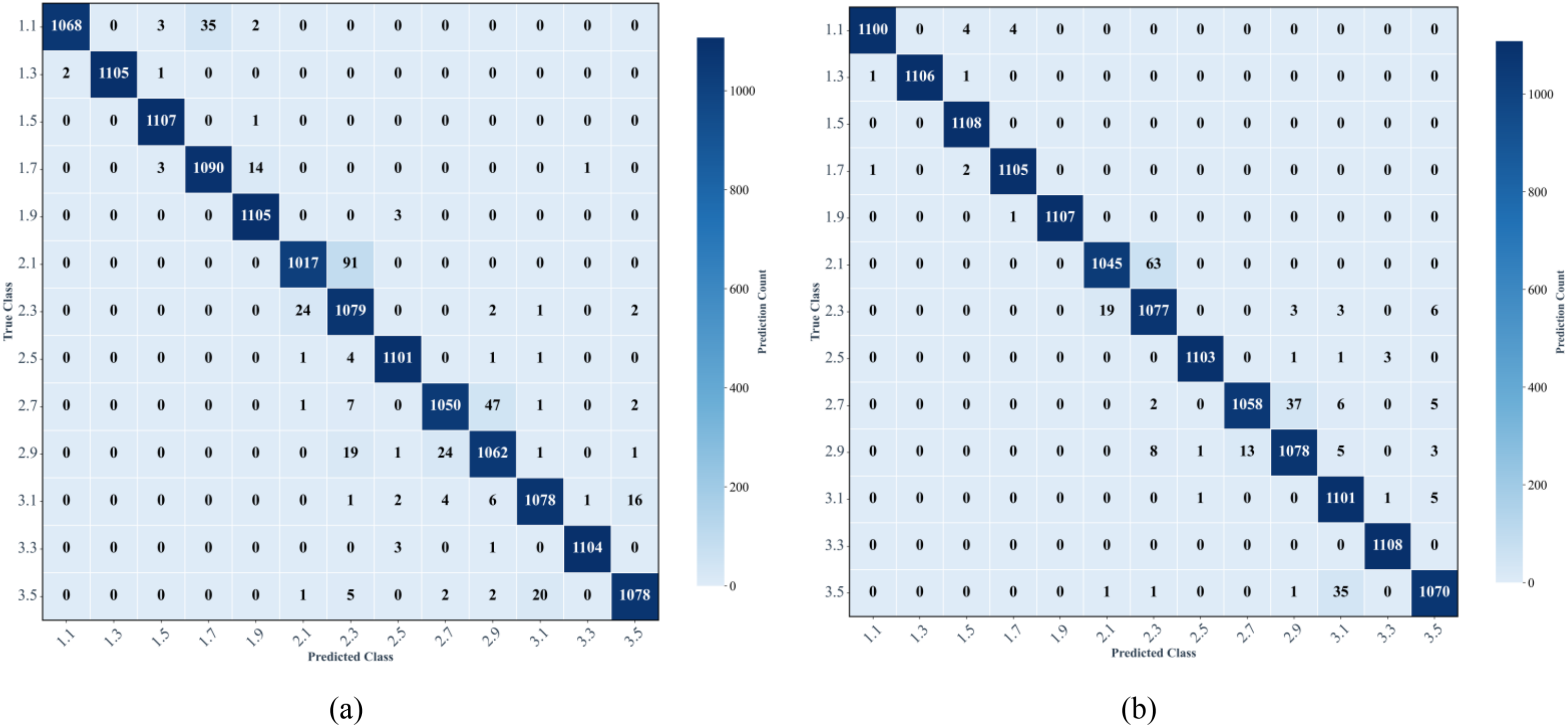
Lresnet50 model confusion matrix and M-Lresnet50 model confusion matrix. **(a)** Confusion matrix of the Lresnet50, **(b)** Confusion matrix of M-Lresnet50.

The confusion matrix is shown in **Figure 10**. This shows that M-Lresnet50 outperformed Lresnet50 in most seedling stages, particularly in stages where the boundaries were blurred. The number of correctly identified samples in stage 1.1 increased by 32, reaching 1,100. Stage 2.1 improved from 1,017 to 1,045, while stage 3.1 rose from 1,078 to 1,101. Furthermore, M-Lresnet50 achieved 100% classification accuracy at stages 1.5 and 3.3. In contrast, Lresnet50 did not reach perfect accuracy for any category. This demonstrates the strong discriminative power of multimodal features at these stages. Further analysis of M-Lresnet50’s misclassification patterns revealed that errors were mainly concentrated between adjacent seedling stages. For instance, the bidirectional confusion of Lresnet50 between stages 2.7 and 2.9 occurred 71 times, whereas with M-Lresnet50 it was reduced to 50 times. Similarly, confusion between stages 2.1 and 2.3 decreased from 115 to 82 instances. Notably, a directional change in confusion was observed for stages 3.1 and 3.5: the number of times stage 3.1 was misclassified as 3.5 decreased from 16 to 5, while the number of times stage 3.5 was misclassified as 3.1 increased from 20 to 35. This led to a decrease in recall for stage 3.5.

In summary, M-Lresnet50 significantly reduced the overall error rate and effectively minimized misclassifications between distant stages, concentrating the remaining errors on adjacent stages. This demonstrates higher stability and robustness. These results suggest that, in the complex task of rice seedling age recognition, multimodal feature fusion can leverage the complementary advantages of different modalities, making it a more reliable and valuable approach than unimodal methods.

### 3.7 Environmental data correlation analysis

**Figure 11:**
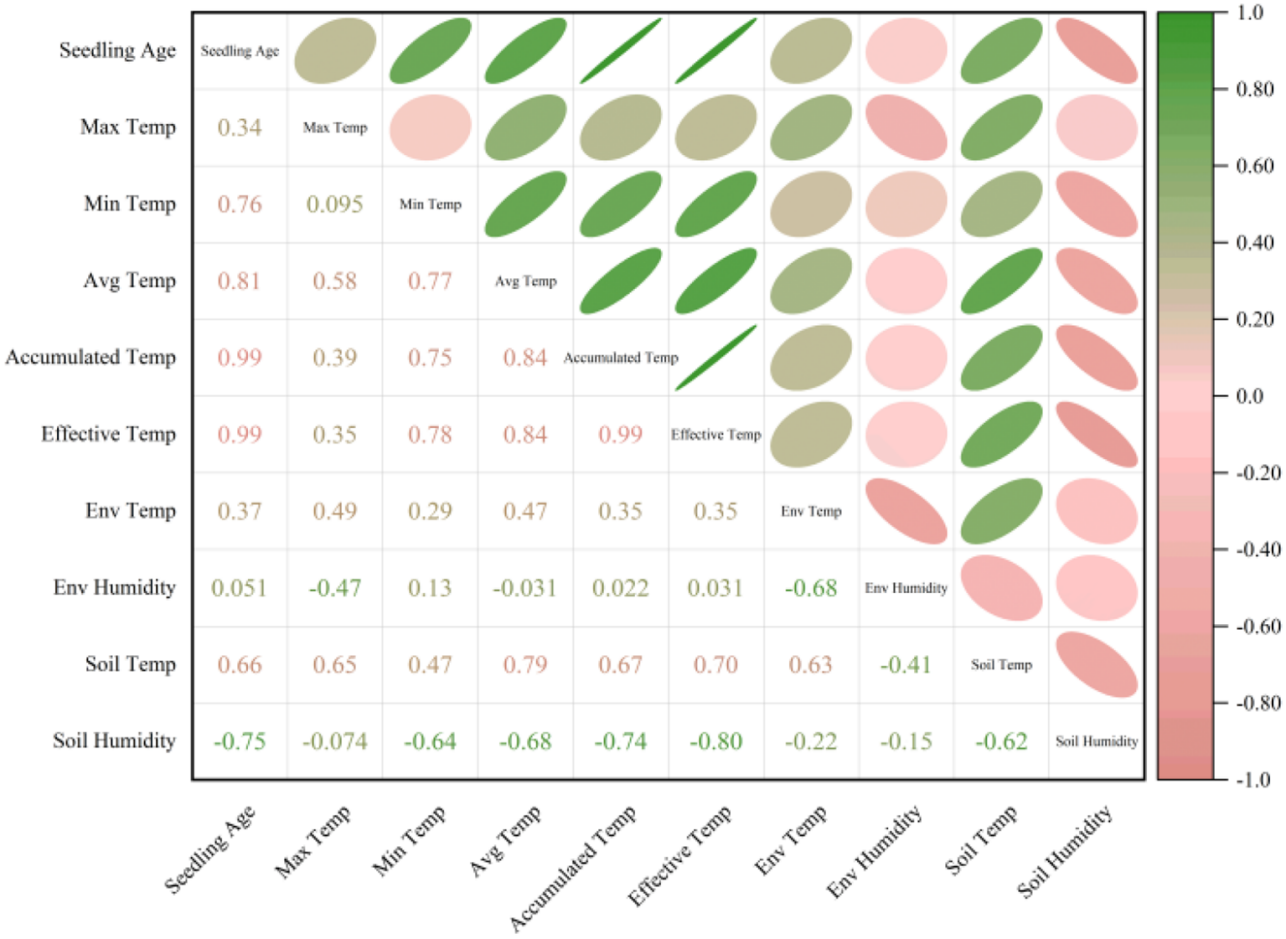
Correlation heatmap.

**Figure 11** shows the Pearson correlation heatmap between rice seedling age and key environmental factors. As can be seen from the figure, seedling age shows a strong positive correlation with various temperature-related indicators. Effective accumulated temperature, accumulated temperature, average temperature and minimum temperature all demonstrate strong correlations, with respective coefficients of 0.99, 0.99, 0.81 and 0.76. This suggests that seedling growth is primarily driven by cumulative heat and stable temperature conditions, with temperature accumulation playing a dominant role in seedling development. In contrast, the correlation between seedling age and maximum temperature is relatively low at 0.34, suggesting that high temperatures have limited explanatory power for seedling development. Furthermore, there is also a certain degree of positive correlation between seedling age and environmental temperature as well as soil temperature, with correlation coefficients of 0.37 and 0.66 respectively. This suggests that suitable environmental and soil temperatures can promote rice growth. However, a significant negative correlation was observed between seedling age and soil moisture (r = -0.75), indicating that excessively high soil water content may inhibit growth. This phenomenon may be related to root hypoxia and impaired nutrient absorption caused by waterlogged soil. Overall, rice seedling age depends primarily on the accumulation and stability of temperature factors, while also being regulated by soil and environmental temperature. Accumulated temperature and effective accumulated temperature are key indicators of changes in seedling age, while soil moisture acts as an important negative regulatory environmental factor. These results provide a quantitative basis for predicting and managing seedling age based on environmental factors.

**Figure 12:**
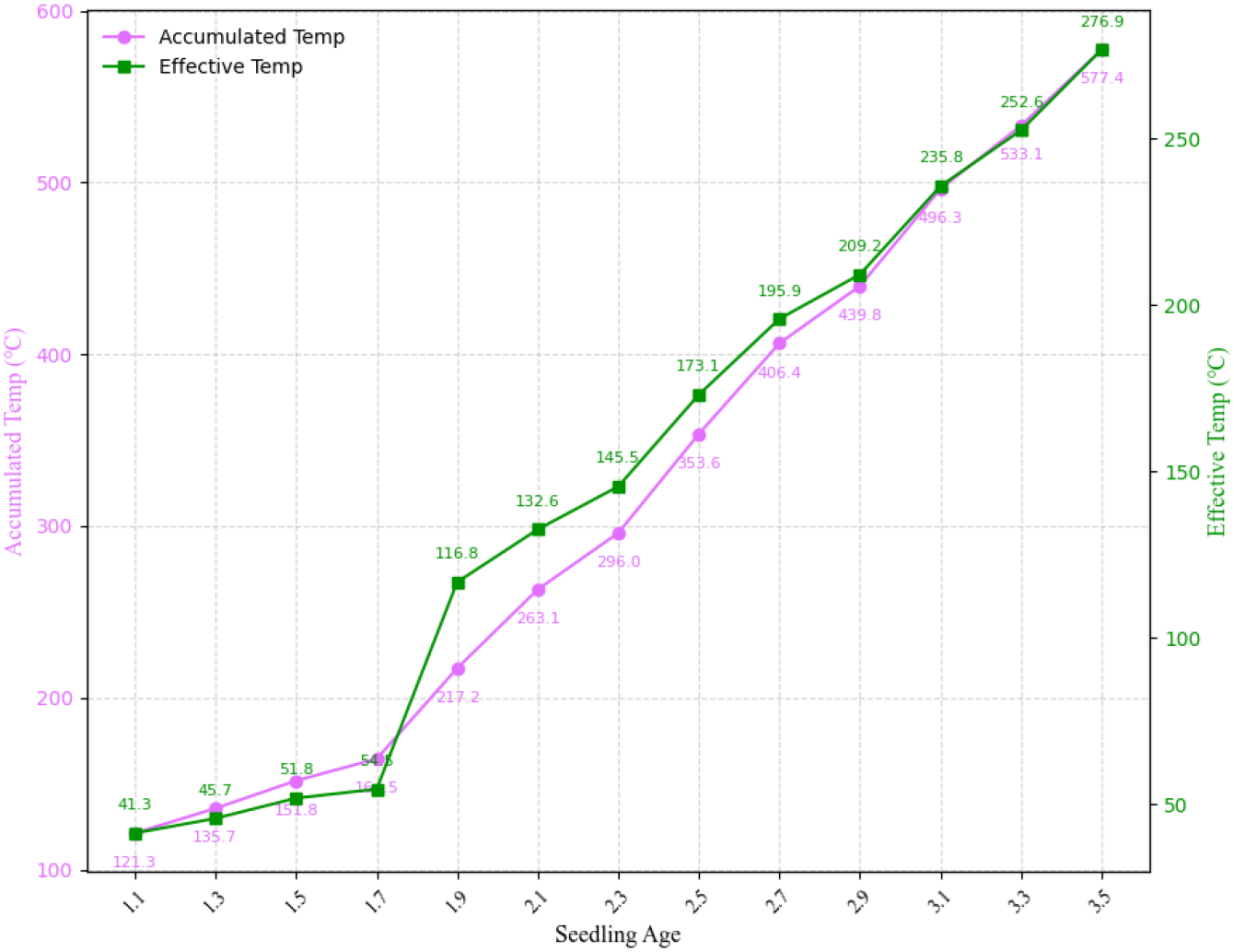
Variation trend of rice seedling age with accumulated temperature and effective accumulated temperature.

**Figure 12** illustrates the dynamic relationship between seedling age and accumulated temperature and effective accumulated temperature, to further analyze the importance of these two indicators in the rice growth process. Overall, seedling age increases gradually with the accumulation of both temperature indices, exhibiting a significant positive correlation. During stages 1.1-1.7, both temperature indices increase relatively slowly. As the seedlings enter the 1.9-3.1 stage, the temperature curve slopes increase significantly and growth accelerates. This indicates that this period is critical for rice development and highly sensitive to temperature conditions. Subsequently, during stages 3.3-3.5, the growth curves begin to level off, suggesting that seedling growth is stabilizing. In summary, both accumulated temperature and effective accumulated temperature effectively reflect the changing patterns of rice seedling age. Effective accumulated temperature is more closely related to seedling age, indicating its higher application value in predicting seedling age and characterizing the growth process.

### 3.8 Model generalization ability verification

**Figure 13:**
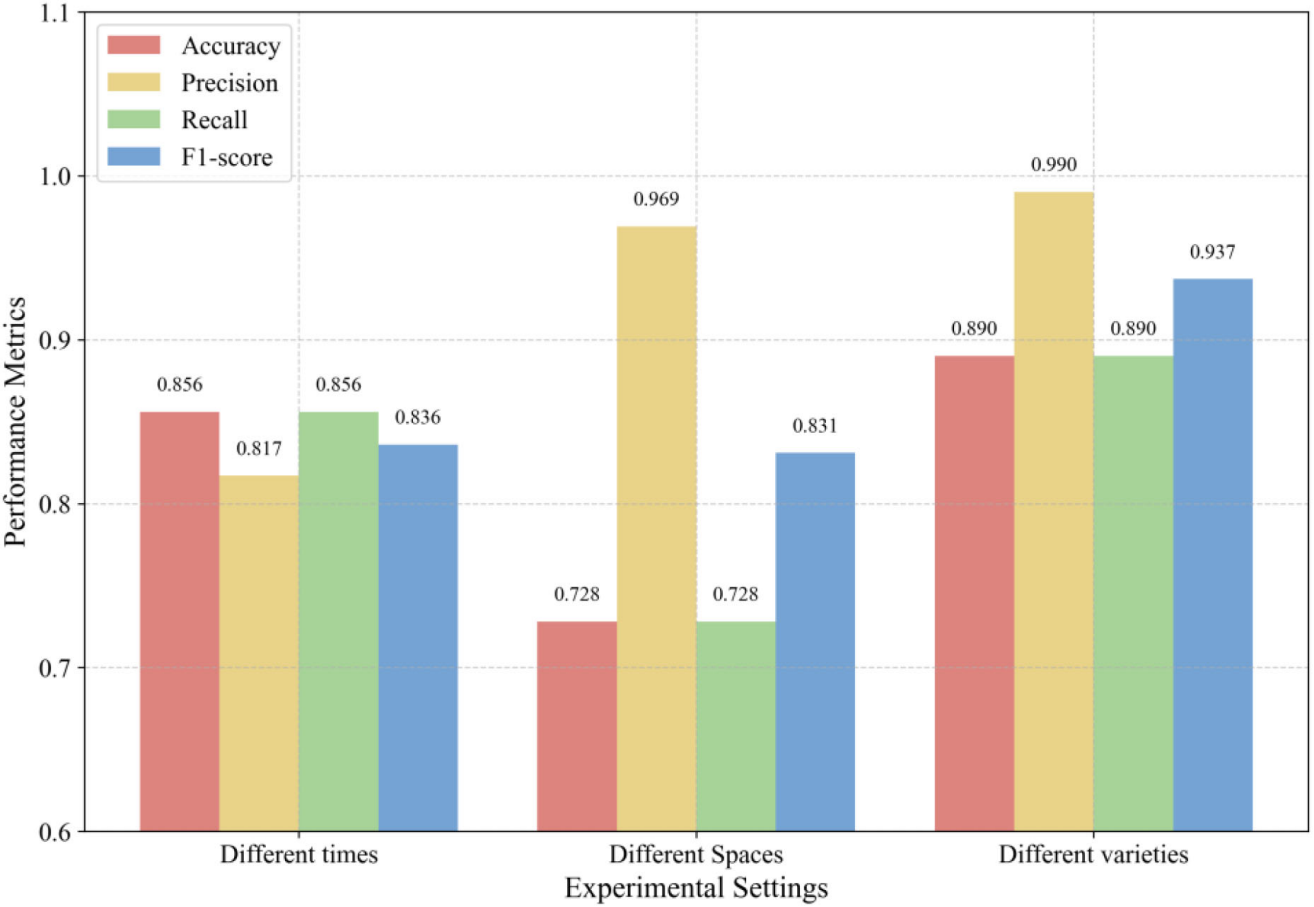
M-Lresnet50 model generalization capability.

To evaluate the robustness and practical potential of the proposed model comprehensively, systematic generalization ability tests were conducted. The results of these experiments are presented in **Table 11** and **Figure 13**. In terms of temporal generalization, the M-Lresnet50 model demonstrated stable performance, achieving an accuracy of 0.856 and an F1 score of 0.836. In comparison, Lresnet50 achieved an accuracy of 0.812. This indicates that the multimodal model can better adapt to climatic variations across different years. The slight decline in performance was primarily attributed to domain shift caused by uncontrollable environmental variables between growing seasons. This reflects the stability and reproducibility of the visual–environmental feature relationships captured by the multimodal model to some extent. In the spatial generalization test, M-Lresnet50 achieved a precision of 0.969 and a recall of 0.728, with an overall accuracy of 0.728, slightly higher than the 0.710 accuracy obtained by Lresnet50. Although Lresnet50 reached a very high precision of 0.998, its lower recall reduced overall robustness under spatial domain shift. These results suggest that while predictions remain reliable in new geographic environments, background differences such as soil color and seedling tray specifications may lead to missed detections. The most notable result came from the cross-variety generalization test. M-Lresnet50 achieved an accuracy of 0.890 and an F1-score of 0.937, whereas Lresnet50 achieved an accuracy of 0.823 and an F1-score of 0.870. Precision reached as high as 0.990, while recall remained high. This demonstrates that the multimodal model does not rely on shallow memorization of specific variety features but effectively captures deeper morphological and environmental growth patterns underlying rice seedling development.

**Table 11:** Model generalization capability.

Experimental settings	Model	Accuracy	Precision	Recall	F1
Different times	Lresnet50	0.812	0.942	0.812	0.872
	M-Lresnet50	0.856	0.817	0.856	0.836
Different Spaces	Lresnet50	0.710	0.998	0.710	0.830
	M-Lresnet50	0.728	0.969	0.728	0.831
Different varieties	Lresnet50	0.823	0.922	0.823	0.870
	M-Lresnet50	0.890	0.990	0.890	0.937

## 4 Discussion

### 4.1 Effectiveness of model design

This study presents a Lresnet50 model that integrates three key modules:RPS, FPN and DCP to significantly improve rice seedling age recognition using a single image modality. Compared with the study by Qin et al. (2023), which divided the entire rice growth period into six stages and used Resnet-50 as the backbone network with an accuracy of 87.33%, our Lresnet50 model achieved an average accuracy of 97.70% in identifying 13 seedling age stages, representing an improvement of 10.37%. This improvement was particularly evident during the ‘transition period’, when morphological differences are subtle, where the proposed model outperformed various mainstream CNN models. Tan et al. (2022) focused on identifying three key stages of seedling growth (BBCH11, BBCH12 and BBCH13) using UAV images of rice. In contrast, this study refined seedling stage recognition to include 13 stages, even those that are morphologically similar, which significantly increases the difficulty of recognition and its practical value. Luo et al. (2023) proposed a method that combines semantic segmentation with the shortest distance algorithm to determine the inclination of rice leaf veins, differentiating between rice plants with six to nine leaves. By contrast, this study classifies rice seedlings from the 1.1-leaf to 3.5-leaf stage. Through targeted structural optimizations, the proposed model achieves highly precise, fine-grained recognition in complex field environments, even when relying solely on visual information.

In recent years, integrating image data with meteorological, environmental and soil data has been shown to enhance the stability and generalization of tasks such as yield prediction and disease detection (Kaur et al., 2023; Yewle et al., 2025). Building on this, we propose the M-Lresnet50 model, which fuses image features with temporal environmental features extracted using an LSTM. This further improves recognition accuracy to 0.9833. Confusion matrix analysis shows that the multimodal model reduces the overall error rate and significantly decreases misclassifications between distant seedling stages, concentrating errors within adjacent stages. This better aligns with the continuous nature of agricultural production and demonstrates the model’s enhanced robustness and rationality in decision-making. Correlation analysis shows that seedling age has a very strong positive correlation with accumulated temperature and effective accumulated temperature (r = 0.99), confirming the dominant role of heat accumulation in rice growth. Conversely, seedling age exhibits a significant negative correlation with soil moisture (r = -0.75), which could reflect the inhibitory effect of higher soil moisture levels on growth during the experimental period. These findings improve the interpretability of the model and provide a theoretical basis for its application in intelligent, predictive irrigation and fertilization decisions.

### 4.2 Generalization ability

Generalization testing was conducted across three dimensions: temporal, spatial, and cross-varietal. The results demonstrate the model’s consistent performance over time, indicating its ability to adapt to data from different years. However, a significant decrease in performance was observed when transferring to different greenhouse environments, suggesting limitations in handling variations in environmental conditions and lighting. The main reasons for this degradation in performance are differences in illumination and shadows, soil background color and texture, variations in seedbed ridge geometry and row spacing, changes in seedling density and inconsistencies in sensor installation and calibration across sites. These factors collectively alter the distribution of image and environmental features, rendering the discriminative representations learnt in the source domain unstable in the target domain. To improve spatial generalization, we will strengthen domain augmentation by introducing more aggressive illumination perturbations and soil style augmentation. We will also incorporate background and geometric variations to broaden the coverage of the training domain. Meanwhile, we will apply feature normalization by standardizing the units of environmental variables, aligning their distributions across sites and improving sensor calibration. When necessary, we will adopt more robust normalization strategies within the network.

Cross-varietal experiments also confirmed the model’s transferability to previously unseen rice varieties, indicating its potential for broader agricultural applications. Overall, the model demonstrated robustness across temporal and cross-varietal domains, but spatial transfer remains challenging. Addressing domain shifts caused by geographic and environmental differences will be a critical focus for future research.

### 4.3 Limitations

Despite the substantial performance gains achieved by the proposed multimodal seedling age recognition method, several limitations remain. First, the model still has certain errors. In addition, the current study relies primarily on top-view images, which mainly capture leaf area and surface morphology, but may not fully reflect vertical leaf growth characteristics such as thickness, curvature, and plant height. Incorporating side-view or multi-view imaging in future work could provide more comprehensive structural information and further improve prediction robustness. Second, the environmental modality only includes basic indicators such as temperature and humidity, failing to capture other key ecological factors like light intensity, soil nutrients, and dynamic water content. This limits the model’s ability to comprehensively represent the crop growth process. Third, the study focuses mainly on greenhouse conditions during the seedling stage in cold regions. Although this reflects actual agronomic practice, model deployment in open-field environments may face additional challenges, such as illumination variability, occlusion, sensor instability, and environmental noise, which require further validation and adaptation.

To address these issues, future research could explore the following directions: Gradually adjust the model to achieve the best effect; Introducing more diverse environmental variables and integrating crop physiological mechanisms to construct multi-dimensional representations; Developing continuous-value seedling age prediction and combining it with crop growth models to enable dynamic monitoring and management.

## 5 Conclusion

This study explores solutions based on multimodal deep learning to address the challenge of accurately identifying subtle and continuous differences in rice seedling stages under complex field conditions. The main conclusions are as follows:

Firstly, a rice seedling stage recognition method based on a lightweight Resnet50 architecture, named Lresnet50, was proposed. The model achieved high-precision recognition of 13 fine-grained seedling stages using a single image modality by incorporating RPS, FPN and DCP modules, with an average accuracy of 0.9770. This significantly outperforms traditional CNN models.

Second, we developed a multimodal fusion model, M-Lresnet50, which integrates image features with environmental time-series information extracted by an LSTM network. This fusion improved recognition accuracy to 0.9833. Correlation analysis revealed a strong association between seedling age and both accumulated temperature and effective accumulated temperature. This validates the critical role of heat accumulation in rice growth and development, while also enhancing the interpretability of the model.

Generalization experiments demonstrated that the model exhibits robustness over time and across different rice varieties. However, its performance declined when transferred to greenhouses in different geographic locations, suggesting that environmental differences and domain shift remain important considerations for practical deployment. Overall, the proposed method offers significant advantages in terms of recognizing the seedling stage in fine detail, fusing multimodal information, and making the model more interpretable. It provides a viable solution for intelligent rice field management, predictive irrigation and fertilization decision-making. Future work could focus on expanding data collection scenarios, enriching environmental modalities, developing continuous value prediction and optimizing model deployment for mobile and edge computing devices.

## Data Availability

The raw data supporting the conclusions of this article will be made available by the authors, without undue reservation.
